# CLDN18.2 antibody design with protein language models: A deep learning optimization framework

**DOI:** 10.1371/journal.pcbi.1014499

**Published:** 2026-08-20

**Authors:** Tao Qu, Lingyan Yuan, Weiran Cui, Jiatian Tang, Zhitong Bing, Xianghong Xu, Jizheng Duan, Qiong Yang, Hui Cai

**Affiliations:** 1 NHC Key Laboratory of Diagnosis and Therapy of Gastrointestinal Tumor, Gansu Provincial Hospital, Lanzhou, Gansu, China; 2 The Institute of Clinical Research and Translational Medicine, Gansu Provincial Hospital, Lanzhou, Gansu, China; 3 Advanced Energy Science and Technology Guangdong Laboratory, Huizhou, China; 4 Fujian Institute of Research on the Structure, Chinese Academy of Sciences, Fuzhou, China; 5 Key Laboratory of Carcinogenesis and Translational Research (Ministry of Education/Beijing), Center of Gastrointestinal Cancer, Peking University Cancer Hospital & Institute, Beijing, China; 6 Department of Computational Physics, Institute of Modern Physics, Chinese Academy of Sciences, Lanzhou, Gansu, China; Indonesia International Institute for Life Sciences, INDONESIA

## Abstract

CLDN18.2 is a promising tumor-specific antigen; however, the development of therapeutic antibodies against it is challenged by the need for simultaneous optimization of affinity and developability. To address this, we present cdrGPT, a deep learning framework based on GPT-2 for de novo generation of complementarity-determining region H3 (CDRH3) sequences. Our approach integrates pre-training on the Observed Antibody Space (OAS) database with structural templating derived from the known antibody zolbetuximab. Generated sequences were iteratively refined through rejection sampling and fine-tuned against a multi-parameter objective function encompassing predicted affinity and MHC class II binding risk. From an initial set of 50,000 sequences, this screening pipeline yielded 313 high-confidence candidates. Subsequent analysis using evolutionary scale modeling 2 (ESM2) embeddings, principal component analysis (PCA), and clustering revealed three structurally distinct clusters, with intra-cluster cosine similarities exceeding 0.99. Validation of seven representative sequences from the dominant cluster using AlphaFold3 confirmed high structural fidelity to the zolbetuximab template, demonstrating a root mean square deviation (RMSD) of 1.331 Å for the CDRH3 loop and positional deviations of less than 0.4 Å for key paratope residues. These results indicate that the designed variants preserve the core binding mode of the parent antibody. This study establishes a feasible pipeline for integrating AI-generated CDRH3 loops into functional antibody scaffolds, providing a foundation for the accelerated development of therapeutics targeting CLDN18.2 and other clinically relevant antigens.

## Author summary

Gastric cancer remains a major cause of cancer-related mortality worldwide, and effective treatment options are still limited for many patients. Claudin 18.2 (CLDN18.2) has emerged as a promising therapeutic target, as highlighted by the clinical success of zolbetuximab. However, the development of improved therapeutic antibodies remains challenging because conventional antibody engineering is often time-consuming, labor-intensive, and difficult to optimize across multiple properties simultaneously. In this study, we developed cdrGPT, an artificial intelligence framework for designing the CDRH3 region of antibodies targeting CLDN18.2. CDRH3 is a highly variable loop in the antibody heavy chain and often plays a central role in antigen recognition. Our approach integrates antibody language modeling with reinforcement learning-based optimization to generate candidate sequences while considering multiple objectives related to predicted binding and developability. The generated candidates were further assessed using computational structural modeling and property-based screening. Our results suggest that cdrGPT can produce diverse and novel CDRH3 sequences within a fixed antibody scaffold and help prioritize candidates with favorable predicted properties. Although experimental validation will be needed, this work provides a computational strategy for antibody optimization and illustrates the potential of AI-guided sequence design to support therapeutic antibody discovery.

## 1. Introduction

Gastric cancer continues to impose a major global disease burden, with especially high incidence in East Asia, Central and Eastern Europe, and South America [1,2]. China is disproportionately affected, accounting for approximately 37.0% of global new gastric cancer cases and 39.4% of gastric cancer deaths in 2022 [3]. Although systemic chemotherapy remains an important treatment option for advanced gastric cancer, the overall prognosis remains poor even in contemporary treatment settings, and long-term survival is still limited [2,4]. Together, these data underscore the need for more effective therapeutic approaches.

Molecularly targeted therapy has become a major focus of gastric cancer research. Current efforts have concentrated on targets and pathways such as human epidermal growth factor receptor 2 (HER2), vascular endothelial growth factor receptor (VEGFR), epidermal growth factor receptor (EGFR), mammalian target of rapamycin (mTOR), and mesenchymal-epithelial transition factor (MET) [4–6]

In parallel with advances in antibody engineering, deep-learning methods have progressively reshaped computational strategies for protein and antibody sequence analysis. Earlier architectures, including convolutional neural networks (CNNs) and multiscale CNN-based models, have been effective for extracting local and multiscale sequence features and have supported tasks such as sequence classification and property prediction. More recently, attention-based architectures have become increasingly prominent because they are well suited to capturing long-range dependencies and global contextual relationships within biological sequences. This methodological progression provides a rationale for adopting Transformer-based generative models for antibody sequence design, particularly when the objective is to model context-dependent residue relationships during CDRH3 sequence generation and optimization.

Claudin 18.2 (CLDN18.2), a tight-junction molecule, is predominantly expressed in gastric mucosa and is largely absent from most normal tissues. Its aberrant expression in gastric cancer and selected other solid tumors has made it an attractive target for therapeutic development [7,8].

Zolbetuximab (Vyloy) is a first-in-class chimeric monoclonal antibody targeting CLDN18.2 and is approved, in combination with fluoropyrimidine- and platinum-containing chemotherapy, for the first-line treatment of HER2-negative, CLDN18.2-positive, locally advanced unresectable or metastatic gastric or gastroesophageal junction adenocarcinoma [9]. In the phase III SPOTLIGHT trial, zolbetuximab plus mFOLFOX6 improved median progression-free survival (10.61 vs 8.67 months) and median overall survival (18.23 vs 15.54 months) compared with placebo plus mFOLFOX6 [10]. Together with the GLOW study, these findings establish CLDN18.2 as a clinically validated therapeutic target in gastric cancer [11]. Despite their broad therapeutic value, conventional antibody discovery workflows remain labor-intensive, time-consuming, and inefficient in exploring large candidate spaces while maintaining target specificity and favorable developability profiles [12]. In parallel, deep learning has transformed protein and antibody sequence analysis. Earlier convolutional and multiscale convolutional neural network models were effective for local feature extraction and sequence-level classification or property prediction [13–15], whereas more recent attention-based models are better suited to capturing long-range dependencies and global sequence context [12,16].

Against this background, generative language models provide a flexible framework for learning sequence distributions and exploring candidate sequence space beyond conventional rule-based or screening-driven strategies [12,16]. Here, we developed cdrGPT, a GPT-based framework for CLDN18.2-directed CDRH3 sequence generation and optimization. We first constructed a CLDN18.2-oriented prior model using complementarity-determining region H3 (CDRH3) sequences from the Observed Antibody Space (OAS) database, and then built cdrGPT as a sequence generation and optimization framework with rejection-sampling fine-tuning (RFT)-guided multi-objective prioritization.

## 2. Materials and methods

### Dataset and preprocessing

To construct a prior model capable of learning the sequence space of the CDRH3 domain, we retrieved and acquired antibody sequences from the OAS database [17] on December 12, 2024, and subjected them to standardization using the numbering scheme of the International ImMunoGeneTics Information System (IMGT) [18]. Subsequently, we performed comprehensive sequence curation on the heavy chain data using Pandas software [19], with specific operations as follows: 1. Extract specific CDRH3 sequences from paired sequence entries; 2. Remove sequences containing ambiguous residues (X); 3. Conduct length-based filtering to retain CDRH3 sequences with an amino acid length of 8–14, ensuring they match the 9-amino acid residue framework of the template antibody zolbetuximab to guarantee structural consistency and computational feasibility. It should be noted that the current version of cdrGPT has been specifically optimized for human IgG-like antibodies and is not suitable for nanobody scaffolds.

### Model architecture

**Fig 1:**
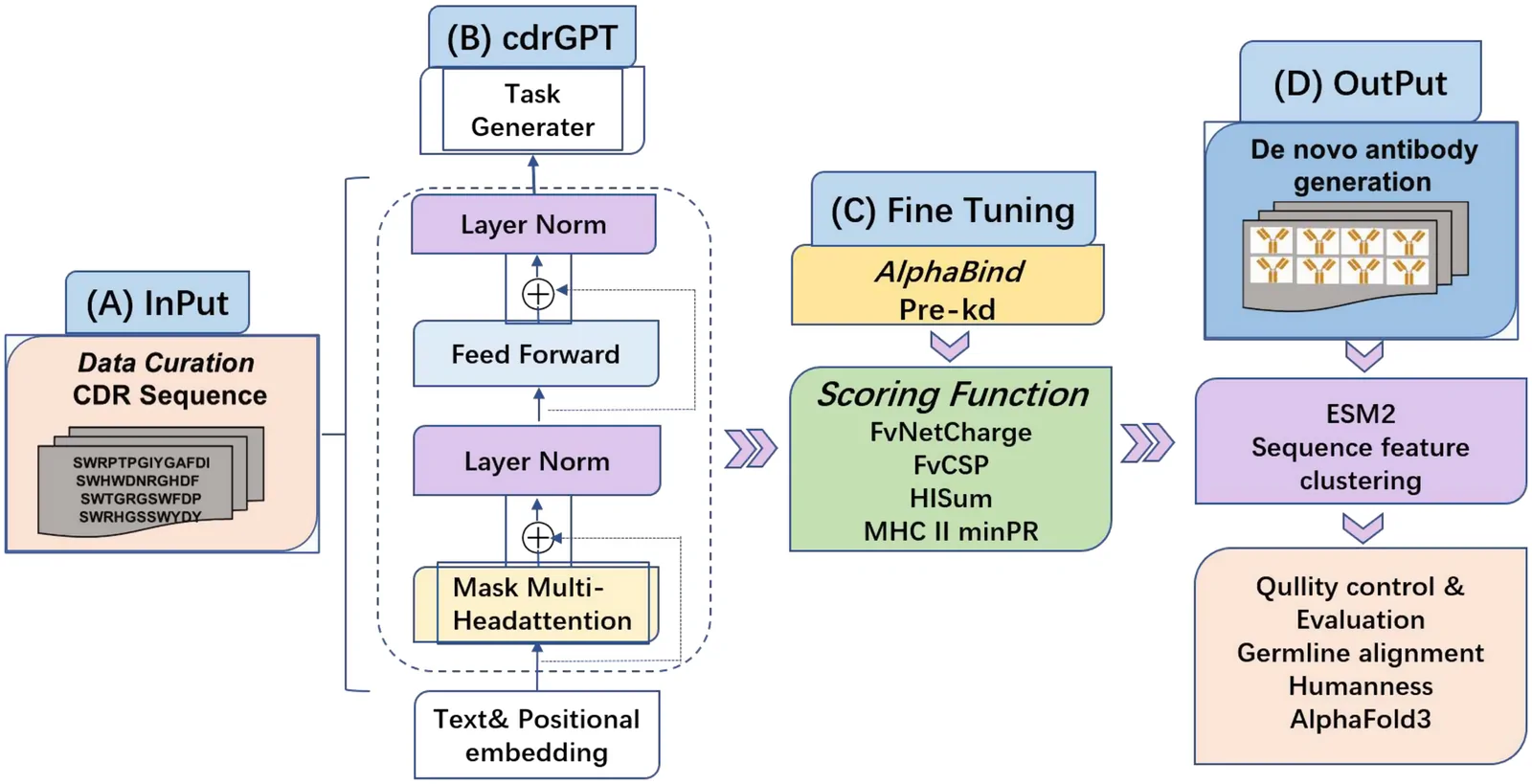
Architecture and workflow of cdrGPT. (A-C) Training workflow of cdrGPT. (A) CDRH3 sequences from the Observed Antibody Space (OAS) were used as model input. (B) During pre-training, a Transformer-based network learned sequence features and long-range dependencies from CDRH3 sequences. (C) During fine-tuning, AlphaBind-predicted dissociation constants (Kd) and multidimensional scoring functions, including FvNetCharge, FvCSP, HISum, and MHC II minPR, were incorporated into model optimization. (D) Workflow for generation and evaluation of candidate antibodies, including ESM2-based feature clustering, germline alignment-based quality control, and AlphaFold3-based structural screening. OAS, Observed Antibody Space.

Fig 1 presents a schematic diagram of the entire workflow. We pre-trained a Transformer decoder model using CDRH3 sequences extracted from the OAS database [17]. This pre-trained model served as the initial parameters for the cdrGPT model. The cdrGPT model was trained via the Rejection Sampling Fine-Tuning (RFT) [20] process, during which scoring functions for the developability of generated antibodies were incorporated to guide the fine-tuning. Ultimately, the fully trained cdrGPT model was used to generate specific CDRH3 sequences targeting the intended receptor protein. These sequences exhibit excellent developability, a property that is crucial for developing highly effective therapeutic antibodies.

### Construction of the prior network

We employed GPT-2 [21], a Transformer-based decoder architecture, as the base model, and the cdrGPT agent adopts the same overall network structure. CDRH3 sequences were tokenized by mapping each amino acid to a unique integer based on its single-letter code, with the addition of special start, end, and padding tokens. The model was trained on the tokenized sequences for next-token prediction, following a causal language modeling approach.

The cdrGPT model is composed of 8 stacked decoder blocks. Each block contains one masked multi-head self-attention layer followed by one feed-forward network (FFN). The self-attention layer outputs a 256-dimensional vector, which is passed to the FFN. The FFN comprises two linear transformations with a gaussian error linear unit (GELU) [22] activation in between, first expanding the dimensionality to 1024 and then projecting it back to 256. The output of the FFN serves as the input to the subsequent decoder block.

The self-attention component follows the standard Transformer decoder formulation described in Attention Is All You Need [23], with causal masking applied so that each token can attend only to preceding tokens.

### Training procedure

Accelerated by four NVIDIA A100 GPUs, the cdrGPT model was trained and evaluated on antibody CDRH3 sequences from the OAS database. Inference was performed on one NVIDIA A100 GPU, and the generation and computational evaluation of 20,000 candidate CDRH3 sequences required approximately 4 h.

### Evaluation system for key physicochemical parameters in antibody engineering of the model

The model was optimized for four key developability attributes: affinity, viscosity, clearance, and immunogenicity. To score generated sequences, full variable region constructs were assembled using a zolbetuximab template. This involved grafting the novel CDRH3 onto the template’s heavy-chain framework, while retaining the native light chain (VL) with its CDRL1 and CDRL3 loops.

For affinity assessment, we calculated the binding score between the assembled VH/VL sequences and CLDN18.2 using the published AlphaBind model [24]. According to this model, a lower score corresponds to a stronger antibody–target affinity.

The viscosity and clearance profiles of the generated antibodies were assessed based on the net charge parameters and hydrophobicity indices of their Fv regions [25]. Prior studies have shown that higher Fv net charge (FvNetCharge) and Fv charge symmetry parameter (FvCSP) values are associated with lower solution viscosity. The clearance rate was primarily influenced by the summed hydrophobicity index (HISum) of CDRL1, CDRL3, and CDRH3. In line with established protocols [25], these parameters were calculated at pH 5.5 by applying the Henderson-Hasselbalch equation to the relevant residues. The HISum was constrained within the range of [0, 4] to modulate clearance [13]. All computed values were benchmarked against those of zolbetuximab.

Explicit humanness scoring using tools such as BioPhi or OASis was not performed in the current study; immunogenicity-related risk was assessed using predicted MHC class II binding, while OAS-derived human antibody pretraining was used to bias sequence generation toward human antibody-like sequence patterns. Immunogenicity was assessed by predicting peptide binding to major histocompatibility complex class II (MHC II) molecules. Using the NetMHCIIpan 4.0 tool [26], we screened a panel of 34 representative human leukocyte antigen (HLA) alleles as per established methodologies. For each 15-mer peptide, the tool provided a percentage rank, inversely indicating the predicted binding affinity to each HLA allele. A higher percentage rank corresponds to weaker binding, thereby suggesting a lower probability of MHC II presentation and subsequent T-cell activation.

For immunogenicity scoring, each CDRH3 sequence was embedded within the Zolbetuximab framework. Template-derived flanking residues were added to its N- and C-termini, generating a complete set of 19 overlapping 15-mer peptides via a sliding window. The minimum percentage rank (minPR) across all resulting peptides and 34 HLA alleles was defined as the sequence’s immunogenicity score. A higher minPR indicates lower predicted immunogenicity.

### Evaluation settings

Step 1: The pre-trained model was first optimized on approximately 637,887 CDRH3 sequences (8–14 amino acids in length) from the Observed Antibody Space (OAS) database. To assess how well the model learned the intrinsic rules governing these sequences, we generated 50,000 novel sequences and compared them against 50,000 sequences randomly sampled from the original training set. A systematic evaluation was then performed by analyzing the consistency of key biochemical property distributions between the generated and the original sequences.

To quantify the quality of the generated sequence set S, we used three key metrics: novelty, uniqueness, and diversity. Novelty was defined as the fraction of sequences in S that were absent from the original training set D, based on exact sequence matching:


$$\begin{array}{c} \text{Novelty}=\;\frac{\left|\left\{\text{x}\in \text{S}:\text{X}\notin \text{D}\right\}\right|}{\left|\text{S}\right|} \end{array}$$ (1)

Uniqueness was defined as the fraction of non-redundant sequences in 𝑆, based on exact sequence identity:


$$\begin{array}{c} \text{Uniqueness}=\;\frac{\left|\text{unique}(\text{S})\right|}{\left|\text{S}\right|} \end{array}$$ (2)

Diversity was defined as the average pairwise Levenshtein distance among sequences in S:


$$\begin{array}{c} \text{Diversity}=\;\frac{\text{2}}{\text{n}(\text{n}-\text{1})}\sum_{\text{x},\text{y}\in \text{S},\text{X}<\text{y}}\text{dist}(\text{x},\text{y}) \end{array}$$ (3)

where dist(x,y) denotes the Levenshtein distance between sequences 𝑥 and 𝑦, and 𝑛 is the number of sequences in the evaluated set. Novelty and uniqueness range from 0 to 1, with higher values indicating a greater proportion of previously unseen sequences and lower redundancy, respectively. Diversity has a lower bound of 0; because it is based on raw Levenshtein distance rather than a normalized score, its upper bound depends on the sequence lengths in the evaluated set rather than a fixed universal range.

Step 2: Generation of CLDN18.2-Specific CDRH3 Sequences

To produce CDRH3 sequences with high specificity for CLDN18.2, we developed a specialized agent model. During generation, key structural residues were preserved to maintain framework integrity: the N-terminal “TR” and C-terminal “W” motifs within the CDRH3 framework of zolbetuximab were fixed. The model was configured with a maximum sequence length of 14 amino acids, yielding 64 candidate CDRH3 sequences per generation step.

Step 3: Generation of CDRH3 Sequences with Multiple Desirable Properties

A fine-tuned model was trained on a multi-property-screened dataset to generate sequences satisfying several property constraints. A scoring function was constructed by integrating metrics including CLDN18.2 affinity (kd_Pred), FvnetCharge, FvCSP, HISum, and predicted MHC-II immunogenicity. Antibodies constructed from CDRH3 sequences generated after two rounds of training were then advanced for subsequent performance comparison and validation.

Step 4: Validation of cdrGPT’s Generalization Capability for Generating CDRH3 Sequences with Desirable Properties

To assess cdrGPT’s generalization in generating functionally specialized CDRH3 sequences, we selected HER2 as a target antigen and applied the framework of trastuzumab (Herceptin) to impose sequence constraints. All generated CDRH3 sequences retained the characteristic N‑terminal “SR” and C‑terminal “Y” residues. During training, 64 sequences were generated per iteration over 3,000 steps. An agent model integrating multiple biophysical property predictors was then developed to produce CDRH3 sequences meeting both HER2‑specificity and multi‑attribute criteria, followed by comparative model analysis.

### Construction of a CLDN18.2-targeted synthetic antibody library

To construct a synthetic antibody library directed against CLDN18.2, 50,000 CDR sequences were systematically retrieved from the antibody repertoire generated using the cdrGPT model. Primary filtering was conducted based on sequence uniqueness and predicted antigen-binding specificity. A subsequent refinement step utilized the predicted antigen-binding affinity (kd_Pred) of the anti-CLDN18.2 monoclonal antibody zolbetuximab as a reference, combined with four critical antibody characteristics: CDRH3 length distribution, net charge, hydrophobicity index, and predicted immunogenicity. This yielded 313 candidate CDRH3 sequences with favorable binding profiles. These selected sequences were then subjected to global multiple sequence alignment via the Clustal Omega algorithm implemented in the R package “msa” (version 1.8.0). Conserved sequence motifs were visualized using sequence logos, which illustrate position-specific amino acid frequencies.

AlphaBind was used as a relative scoring tool to prioritize generated CDRH3 candidates rather than as a predictor of absolute binding affinity. The clinically validated CLDN18.2-binding antibody zolbetuximab was used as a reference baseline, and generated candidates were ranked according to their predicted AlphaBind scores relative to this reference. Because systematic experimental binding data for the generated candidates were not available, AlphaBind scores were not interpreted as calibrated affinity values. Instead, they were combined with developability- and immunogenicity-associated filters, including FvNetCharge, FvCSP, HISum, and predicted MHC class II binding risk, as part of a multi-step computational prioritization pipeline.

### Antibody-antigen interaction

This study was structured as a candidate generation and downstream-screening pipeline rather than as a conventional supervised prediction task. Therefore, evaluation focused on the properties of generated candidates in downstream analyses, rather than on predictive performance under a benchmark-style train/validation/test framework. The generated CDRH3 sequences showed high similarity to the zolbetuximab reference sequence in the embedding-based analysis.

To represent antibody sequence features, we used Evolutionary Scale Modeling 2 (ESM2) [27], a protein language model pre-trained on large-scale protein sequence data. CDRH3 sequences were embedded into a feature space using ESM2, enabling sequence representation and comparison for downstream analysis. Based on these embeddings, a cosine similarity-based clustering analysis was performed to assess the relationship between generated sequences and the zolbetuximab CDRH3 sequence. The generated CDRH3 sequences showed high similarity to the zolbetuximab reference sequence in the embedding-based analysis. Based on these results, LASSO regression was used as a sparse screening approach to prioritize candidate sequences with relatively high similarity-related contribution scores, yielding seven sequences for subsequent structural evaluation. Finally, AlphaFold3 (AF3) on the AF3 server [28] was used for two predictive analyses: prediction of the CLDN18.2 receptor structure and prediction of the interactions between CLDN18.2 and antibody candidates containing the selected CDRH3 sequences.

## 3. Results

### Schematic diagram of the study design

This study focuses on the development of AI-driven CLDN18.2-targeted antibody therapeutics and introduces cdrGPT, a novel model that integrates two core functional modules: a de novo design pre-training module for heavy-chain CDRH3 sequences and an optimization fine-tuning module. This establishes a complete “design-optimization” workflow.

As illustrated in Fig 1, the cdrGPT workflow follows a progressive design. First, in the data preprocessing phase (Fig 1A), CDRH3 sequences were curated from the OAS Database. Rigorous cleaning was performed through homology filtering, length standardization, and outlier removal, followed by format normalization to ensure input data reliability and consistency.

Next, during model construction and feature extraction (Fig 1B), cdrGPT was built on a Transformer architecture incorporating text embedding, positional embedding, masked multi-head attention, feed-forward networks, and layer normalization. This enables efficient extraction of CDRH3 sequence features and captures implicit structure–function correlations, providing a foundation for subsequent antibody generation and property optimization.

In the antibody sequence optimization phase (Fig 1C), the AlphaBind model was used to compute antibody-antigen dissociation constants (kd_Pred). Multi-dimensional scoring functions, including FvNetCharge, FvCSP, HISum, and MHC II scores were integrated to enable iterative refinement of the generated antibody sequences.

Finally, in the quality control and druggability validation phase (Fig 1D), multifaceted validation approaches were applied, including germline gene homology alignment and AlphaFold3-based 3D structure prediction. These steps assess the druggability of the generated CDRH3 sequences, ensuring selected candidates are suitable for further development.

### Learning the CDRH3 sequence space with a pre-trained model

**Fig 2:**
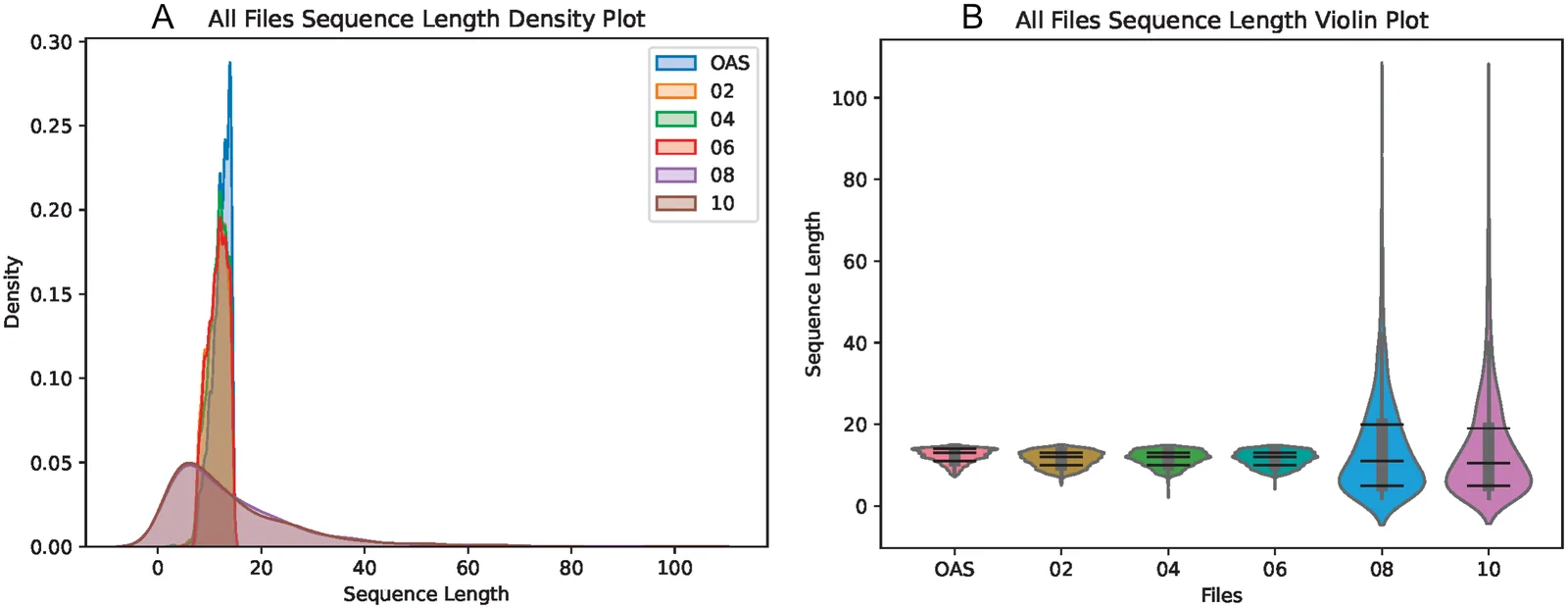
Distributions of amino acid sequence lengths across datasets. (A) Density distributions of sequence lengths across different datasets. (B) Violin plots showing the sequence length distributions in each dataset.

To enable the cdrGPT model to effectively learn the sequence space of the CDRH3 sequences, we systematically curated a training dataset from the OAS database [17]. After comprehensive data processing and analysis, a total of 637,887 non-redundant and unique CDRH3 sequences were obtained, and the density curve and violin plot of their length distribution are detailed in Fig 2 (S1 Fig).

The aforementioned sequences were used as training data to conduct targeted training on the cdrGPT pre-trained model, aiming to enable it to fully learn the characteristics of the CDRH3 sequence space within this specific length range, thereby enhancing the model’s predictive and generalization capabilities in this space. Meanwhile, to optimize model performance, a systematic analysis of hyperparameters was performed to determine the optimal configuration, and the detailed process and results of this analysis are provided in S1 Table and S2 Fig.

### Minimize the model’s loss function

**Fig 3:**
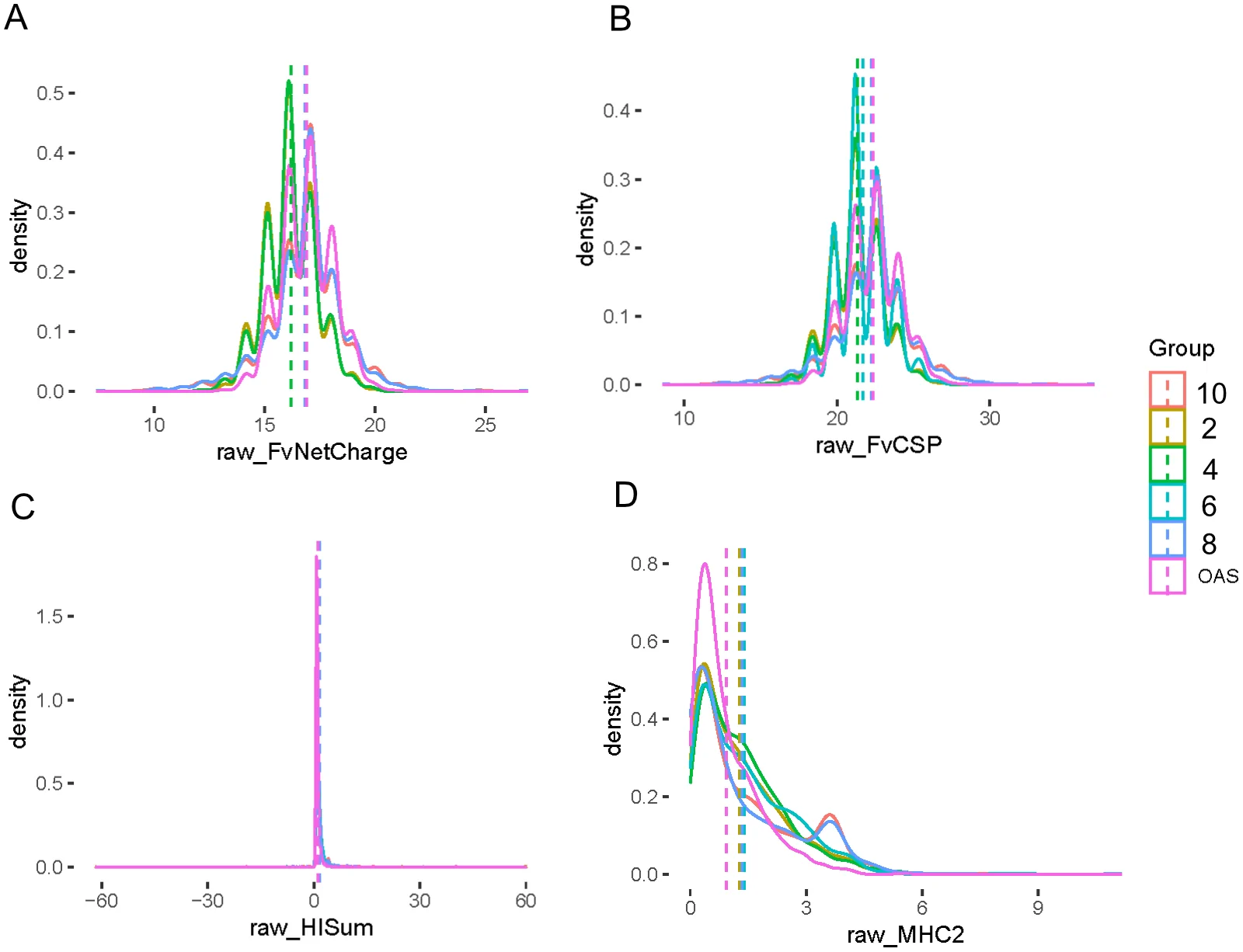
Density distributions of developability-related variables across experimental groups. Density distributions of (A) FvNetCharge, (B) FvCSP, (C) HISum, and (D) MHC II minPR across experimental groups. FvNetCharge, variable fragment net charge; FvCSP, variable fragment charge symmetry parameter; HISum, hydrophobicity index sum; MHC, major histocompatibility complex.

Fig 3 compares the CDRH3 sequence length distributions of cdrGPT-generated sequences across training epochs 2, 4, 6, 8, and 10 with 1,000 natural CDRH3 sequences randomly sampled from the OAS database. This comparison was used to help identify the optimal model checkpoint by assessing how closely the generated length profiles resembled those of natural antibodies.

Epoch 6 was identified as the optimal checkpoint, where the model converged stably without overfitting. Sequences generated at this epoch showed a length distribution concentrated within the 10–14 residue range, closely matching the physiological characteristics of natural CDRH3 regions. No statistically significant differences were observed between Epoch 6 sequences and natural OAS sequences in key performance metrics (P > 0.05).

In contrast, sequences from Epochs 8 and 10 displayed signs of overfitting. The model learned noise from the training data, producing abnormally long sequences (>100 residues) that lacked natural structural feasibility. Therefore, all subsequent analyses and the candidate antibody library were constructed exclusively using sequences generated with the Epoch 6 weights.

We further analyzed the density distributions of four developability-related variables—FvNetCharge, FvCSP, HISum, and MHC II, across the generated (epochs 2,4, 6, 8, and 10) and natural sequence groups (Fig 3). Compared to the natural OAS set, all training-phase samples showed similar distribution shapes but with generally lower peak values, indicating an overall reduction in these variable scores. Distributions for FvNetCharge, FvCSP, and MHC II were multimodal and dispersed, with secondary peaks near the main intervals (Fig 3A, 3B and 3D), whereas HISum displayed a sharp unimodal distribution (Fig 3C).

### Model performance evaluation

To evaluate the effect of sequence optimization, we analyzed key developability- and immunogenicity-related properties in 50,000 CDRH3 sequences generated by three models: the pretrained model (Pre) and two fine-tuned variants (FT1 and FT2).

**Fig 4:**
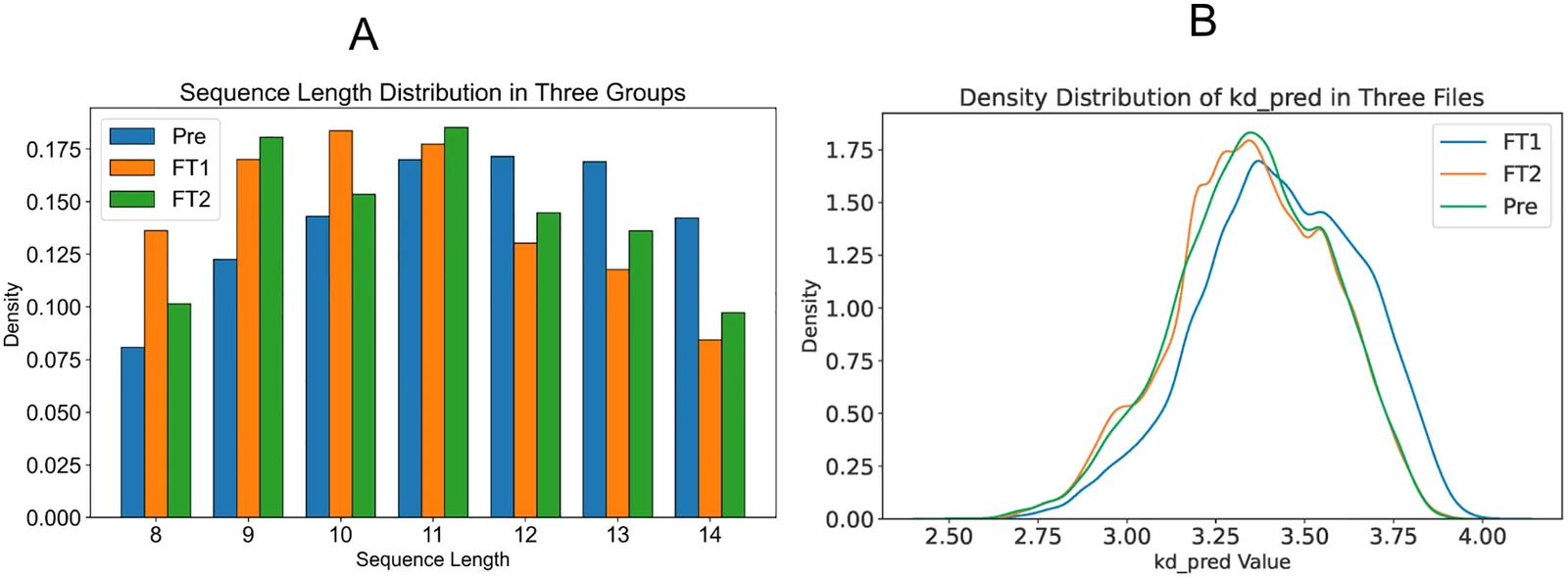
Sequence length and predicted affinity in three datasets. (A) Histogram of sequence lengths. (B) Density distribution of predicted dissociation constant (Kd_pred). Pre_train, pre-trained model dataset; FT1, fine-tuned model 1 dataset; FT2, fine-tuned model 2 dataset.

As shown in Fig 4A, the length distributions of sequences from all models exhibited periodic peaks predominantly within the characteristic CDRH3 residue range, with overall patterns remaining highly consistent despite minor variations in peak intensity.

In affinity predictions, the density distribution of kd_Pred values showed a clear unimodal shape centered near 3.25 (Fig 4B). FT2 (yellow curve) displayed a lower peak and narrower spread compared to FT1 and Pre, indicating reduced variability and more consistent affinity predictions. FT2 also demonstrated superior concentration in several structural and biophysical features.

**Fig 5:**
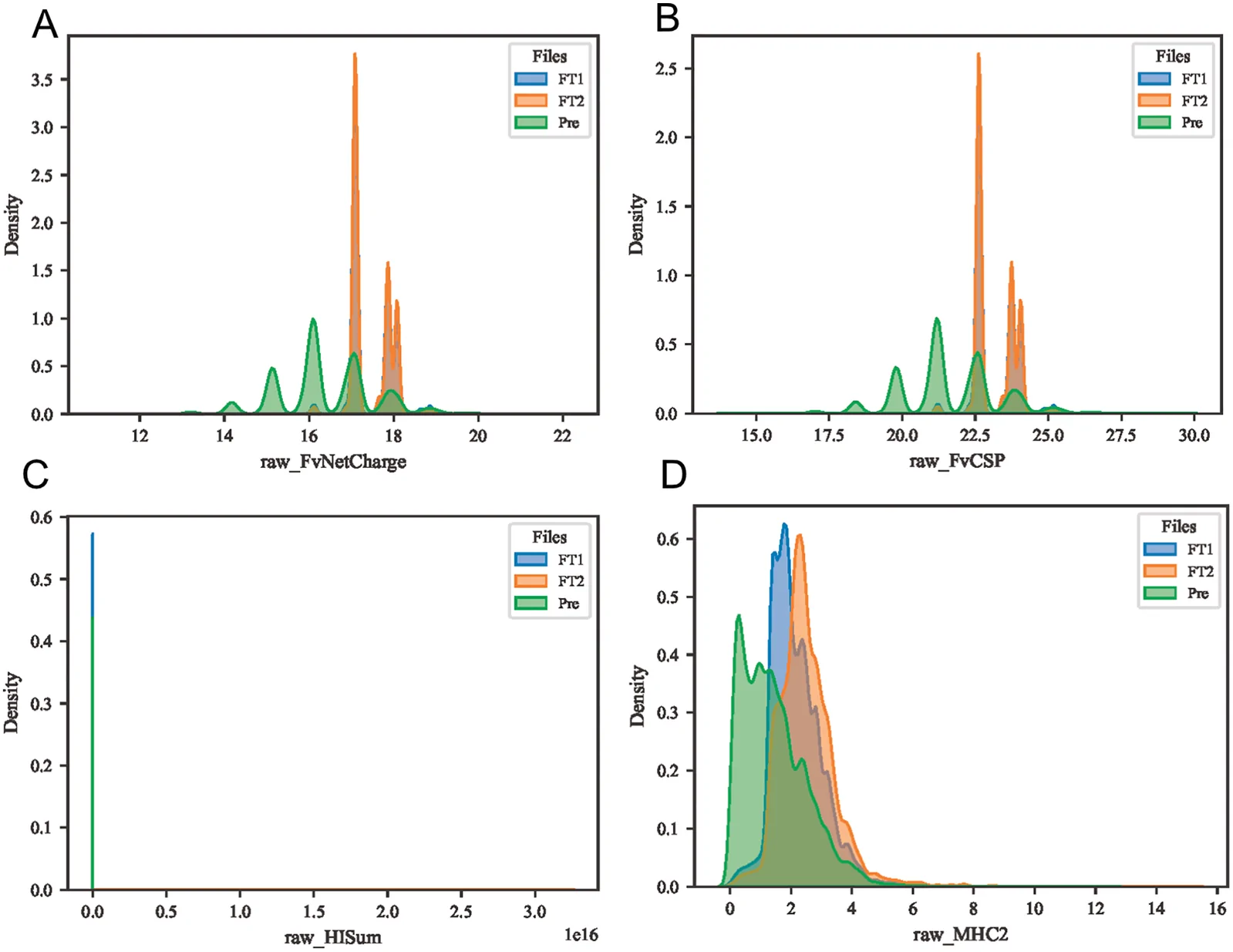
Distributional comparison of representative sequence properties before and after task-specific optimization. Density distributions are shown for CDRH3 sequence sets generated from the pretrained model before optimization (Pre) and after subsequent fine-tuning/rejection-sampling optimization stages (FT1 and FT2). The compared metrics include FvNetCharge (A), FvCSP (B), HISum (C), and predicted MHC II binding risk (D). The observed distributional shifts indicate that fine-tuning/RFT reshaped the generated sequence space with respect to developability- and immunogenicity-associated properties.

As shown in Fig 5, the distributions of these properties differed across the Pre, FT1, and FT2 sequence sets, indicating that fine-tuning/RFT altered the generated sequence space. For FvNetCharge (Fig 5A), FT2 showed a tight unimodal distribution between 16–18, reflecting improved consistency. Its FvCSP values (Fig 5B) were sharply concentrated between 22.5-23.5. While HISum values for FT2 were slightly dispersed (Fig 5C), its distribution was notably tighter than the broad spread of FT1. In contrast, Pre showed excessive aggregation and FT1 exhibited high dispersion, both suggesting suboptimal control. For MHC II scores (Fig 5D), FT2 produced a pronounced peak in the 0–4 range, outperforming the multimodal and broad distributions of FT1 and Pre.

Collectively, FT2 achieved more focused distributions and greater regulatory stability across multiple key attributes compared to FT1 and Pre. This demonstrates the model’s enhanced ability to generate CDRH3 sequences with controlled and consistent feature values.

To assess generalization, we fine-tuned the model for HER2 targeting (parameters in S2 Table and S3 Fig) and evaluated sequence uniqueness, novelty, and diversity in S3 Table.

We compared sequences generated by the pretrained model and two fine-tuned models with a training-set reference baseline using uniqueness, novelty, and diversity metrics. The pretrained model showed near-maximal uniqueness and novelty (1.000 and 0.999, respectively) and high diversity (average pairwise Levenshtein distance = 9.54). The training-set reference baseline showed high uniqueness and diversity within the sampled reference set but zero novelty, as expected, because all sequences were derived from the training data. The two fine-tuned models maintained high uniqueness and novelty but showed reduced diversity (4.741 and 5.459), suggesting that task-specific fine-tuning preserved the ability to generate non-redundant, training-set-unmatched sequences while constraining the explored sequence space.

**Fig 6:**
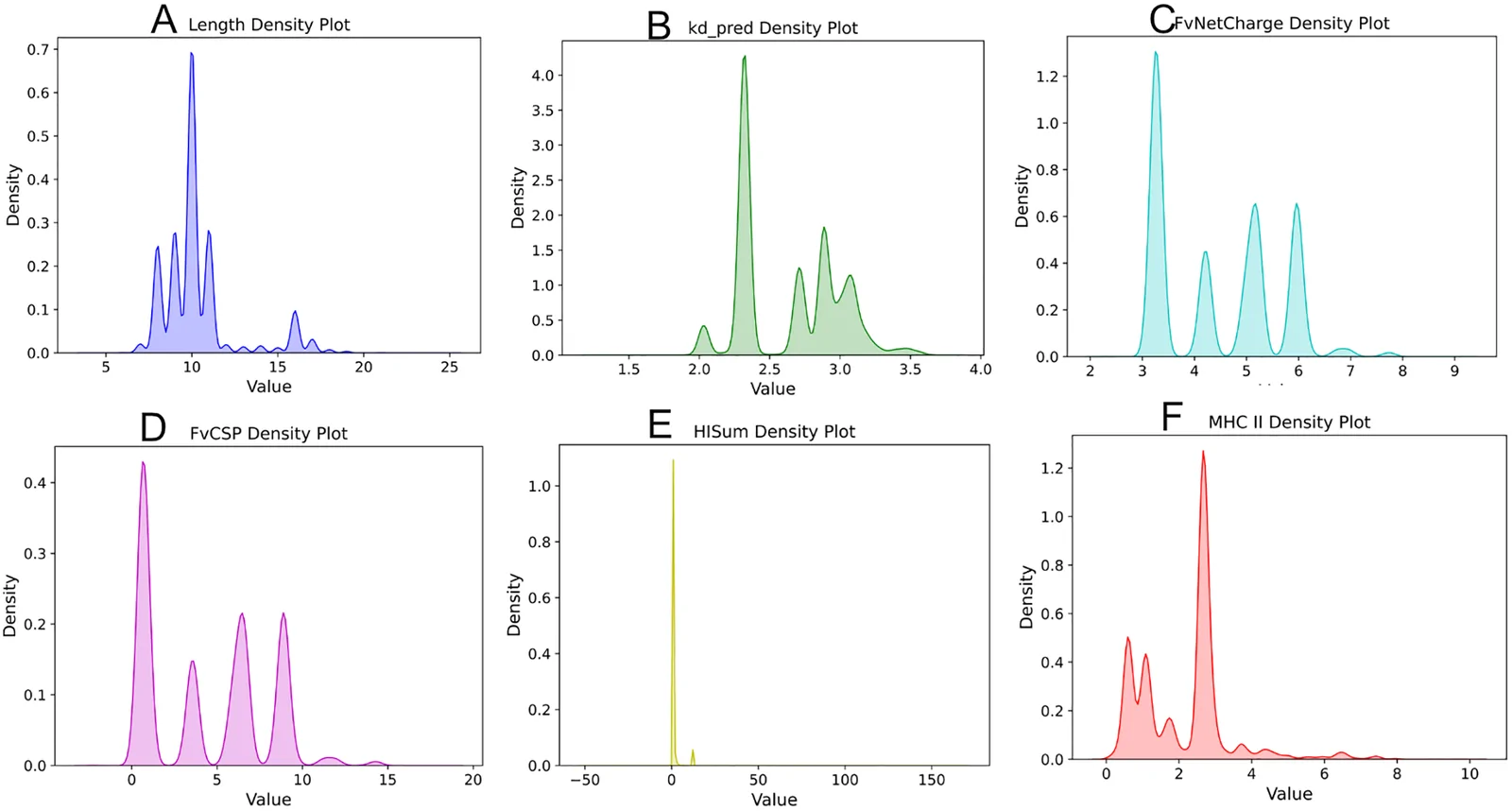
Sequence length and developability-related properties of generated HER2-specific antibody sequences. (A) CDRH3 length; (B) predicted dissociation constant (Kd_pred); (C) FvNetCharge; (D) FvCSP; (E) HISum; and (F) MHC II score. Kd_pred, predicted dissociation constant; FvNetCharge, variable fragment net charge; FvCSP, variable fragment charge symmetry parameter; HISum, hydrophobicity index sum; MHC II, major histocompatibility complex class II binding propensity.

We grafted 5,000 generated HER2-targeting CDRH3 sequences onto the trastuzumab framework and evaluated their developability profiles (Fig 6). The distributions of six core properties aligned closely with trastuzumab’s benchmarks: sequence length (10–15 aa; Fig 6A), kd_Pred peak (2.0-2.5; Fig 6B), net charge (2–4; Fig 6C), hydrophilicity (5–10; Fig 6D), HISum (0–50; Fig 6E), and MHC II scores (mostly <2; Fig 6F). This consistent alignment confirms the model’s generalization capability for generating HER2-specific CDRH3 sequences.

To further evaluate the model’s stability and generalization capability, we utilized cdrGPT to generate CDRH3 sequences for three clinical antibodies (adalimumab, bevacizumab, and panitumumab) and analyzed their key biophysical properties. The model exhibited distinct property preferences for each antibody: adalimumab performed well in FvCSP and kd_Pred; bevacizumab showed advantages in FvNetCharge and affinity control; and panitumumab excelled in MHC II scores. These differences indicate that the model retains antibody-specific attributes, suggesting that practical applications should align sequence generation with specific optimization objectives.

**Fig 7:**
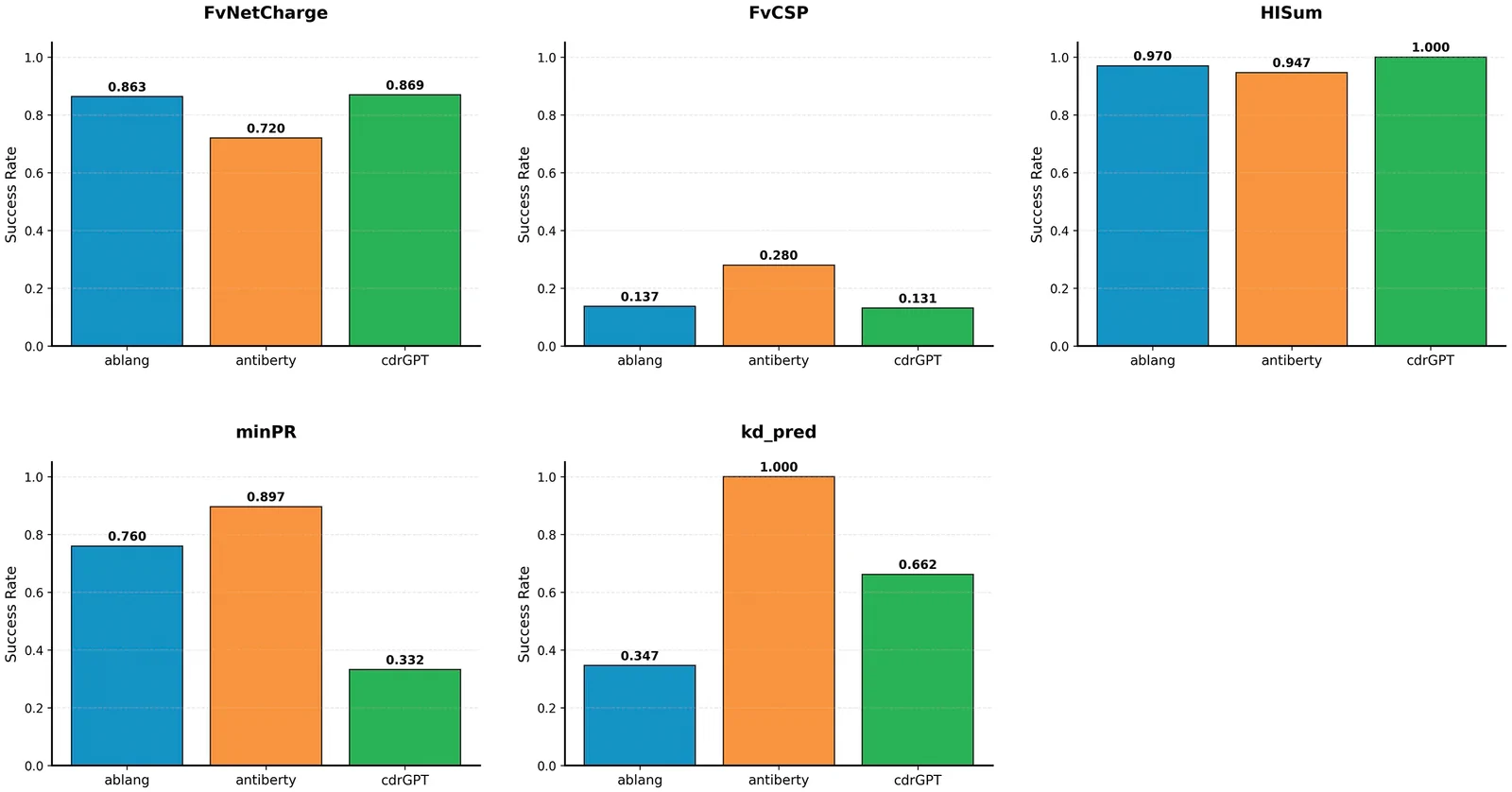
Benchmark comparison of cdrGPT, AbLang, and AntiBERTy under the zolbetuximab-based CDRH3 grafting framework. Three hundred CDRH3 sequences generated by each model were grafted onto the zolbetuximab framework and evaluated using the same biophysical and developability-related criteria. cdrGPT showed higher success rates for HISum and FvNetCharge and retained variable-length CDRH3 generation, whereas its MHC II minPR performance was comparatively weaker than that of AbLang and AntiBERTy.

Additional cross-scenario validation analyses across multiple therapeutic antibody systems are provided in S4 and S5 Figs, supporting the applicability of the pipeline while also revealing antibody-dependent variability. To further contextualize model performance, we compared cdrGPT with AbLang and AntiBERTy under the zolbetuximab benchmark setting using the same CDRH3 grafting and evaluation framework (Fig 7). Under this shared setting, cdrGPT showed the highest HISum success rate and a slightly higher FvNetCharge success rate, while retaining the ability to generate variable-length CDRH3 sequences. However, its performance in MHC II minPR was comparatively weaker than that of the reference models. These results indicate that cdrGPT exhibits a distinct, metric-dependent performance profile rather than uniform superiority across all evaluated measures.

Three hundred CDRH3 sequences generated by cdrGPT, AbLang, and AntiBERTy were grafted onto the zolbetuximab framework and evaluated using the same key biophysical criteria. cdrGPT showed advantages in FvNetCharge and HISum and retained variable-length CDRH3 generation, whereas its performance in MHC II minPR was relatively weaker than that of the comparative models.

### Screening of candidate antibody sequences with development potential

Zolbetuximab was used as the reference antibody for candidate selection. Newly generated sequences were first ranked according to their predicted AlphaBind scores relative to zolbetuximab, and were then filtered using zolbetuximab-guided developability criteria (FvNetCharge < 18.1, FvCSP > 22.6, HISum ∈ [0,4], and MHC II > 1.29). From the FT2 dataset, 50,000 CDRH3 sequences were evaluated via template-guided splicing, resulting in 313 unique antibodies satisfying all criteria.

**Fig 8:**
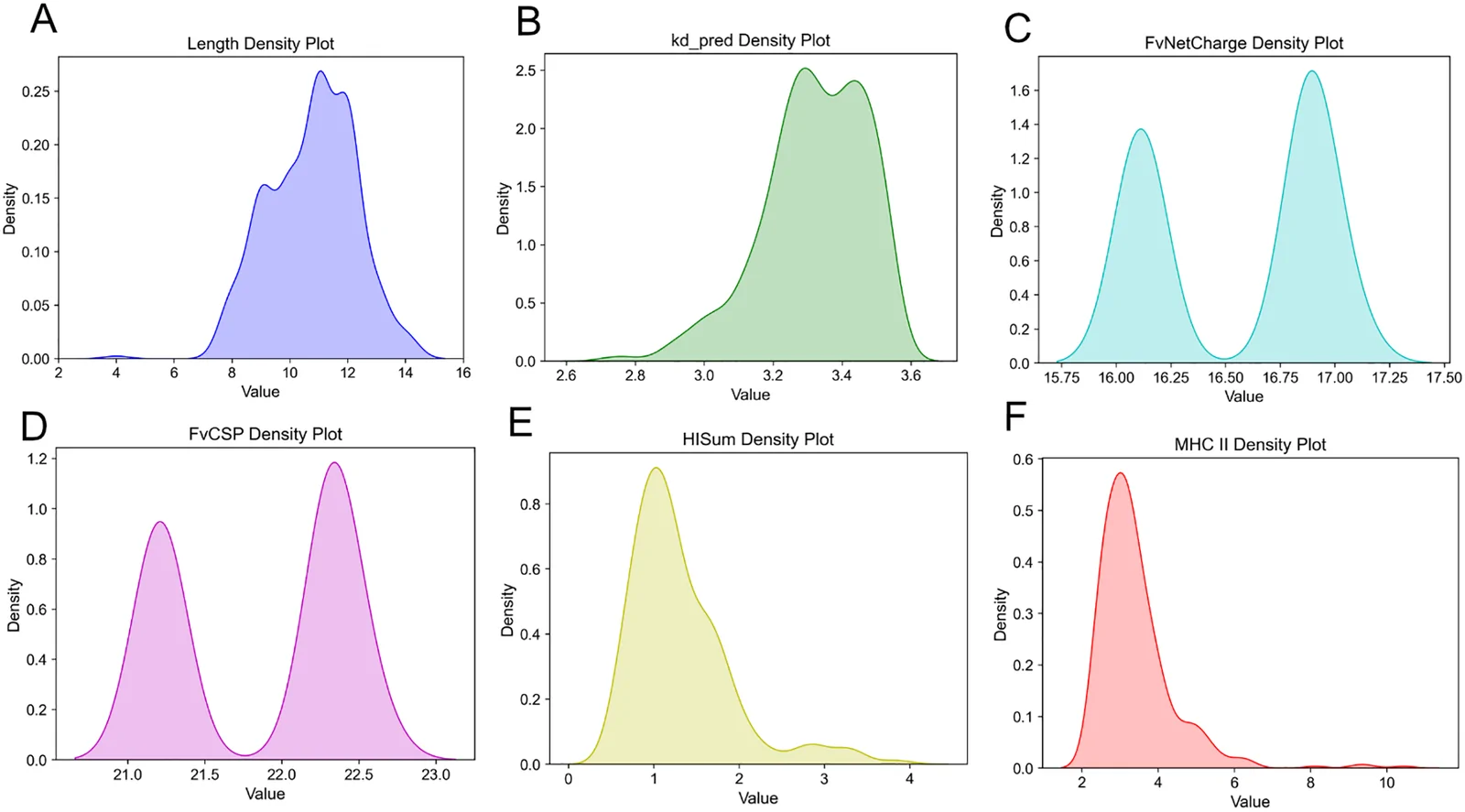
Sequence length and physicochemical properties of the 313 screened sequences. (A) CDRH3 length distribution of the 313 screened sequences. (B) Kd_pred; (C) FvNetCharge; (D) FvCSP; (E) HISum; and (F) MHC II. Kd_pred, predicted dissociation constant; FvNetCharge, variable fragment net charge; FvCSP, variable fragment charge symmetry parameter; HISum, hydrophobicity index sum; MHC II, major histocompatibility complex class II.

The density distributions of CDRH3 lengths and key biochemical attributes were systematically analyzed for the selected sequences. As shown in Fig 8, six core features were examined. CDRH3 length exhibited a clear unimodal distribution centered near 10 residues (Fig 8A), reducing sequence variability due to length heterogeneity. The kd_Pred values formed a symmetrical unimodal distribution between 3.0 and 3.4 (Fig 8B), suggesting uniform solubility or stability. FvNetCharge converged within 16.25-17.00 as a symmetrical unimodal distribution (Fig 8C), indicating consistent charge profiles. FvCSP displayed a bimodal distribution with two peaks of similar magnitude in the range of 21.5-22.5 (Fig 8D), implying conserved structural characteristics. HISum showed a unimodal distribution centered at 1.0, reflecting homogeneous hydrophobicity, while MHC II formed a symmetrical unimodal distribution between 2.0 and 4.0, denoting comparable MHC II binding potential across sequences.

The high-density clustering and strong feature consistency establish a reliable foundation for subsequent functional validation and antibody development, confirming the efficacy of the screening strategy in ensuring sequence uniformity.

### ESM2 feature clustering can rapidly identify antibody subsets with similar functions

To efficiently identify antibodies with shared functional traits, we used the Transformer-based protein language model ESM2 to generate contextual feature embeddings for each antibody sequence. These high-dimensional vectors serve as quantitative “digital fingerprints,” capturing evolutionary and functional relationships. Dimensionality reduction via principal component analysis allowed effective clustering of the embeddings, revealing the topological organization of sequences in the ESM2 feature space and establishing a data-driven framework for functional classification.

**Fig 9:**
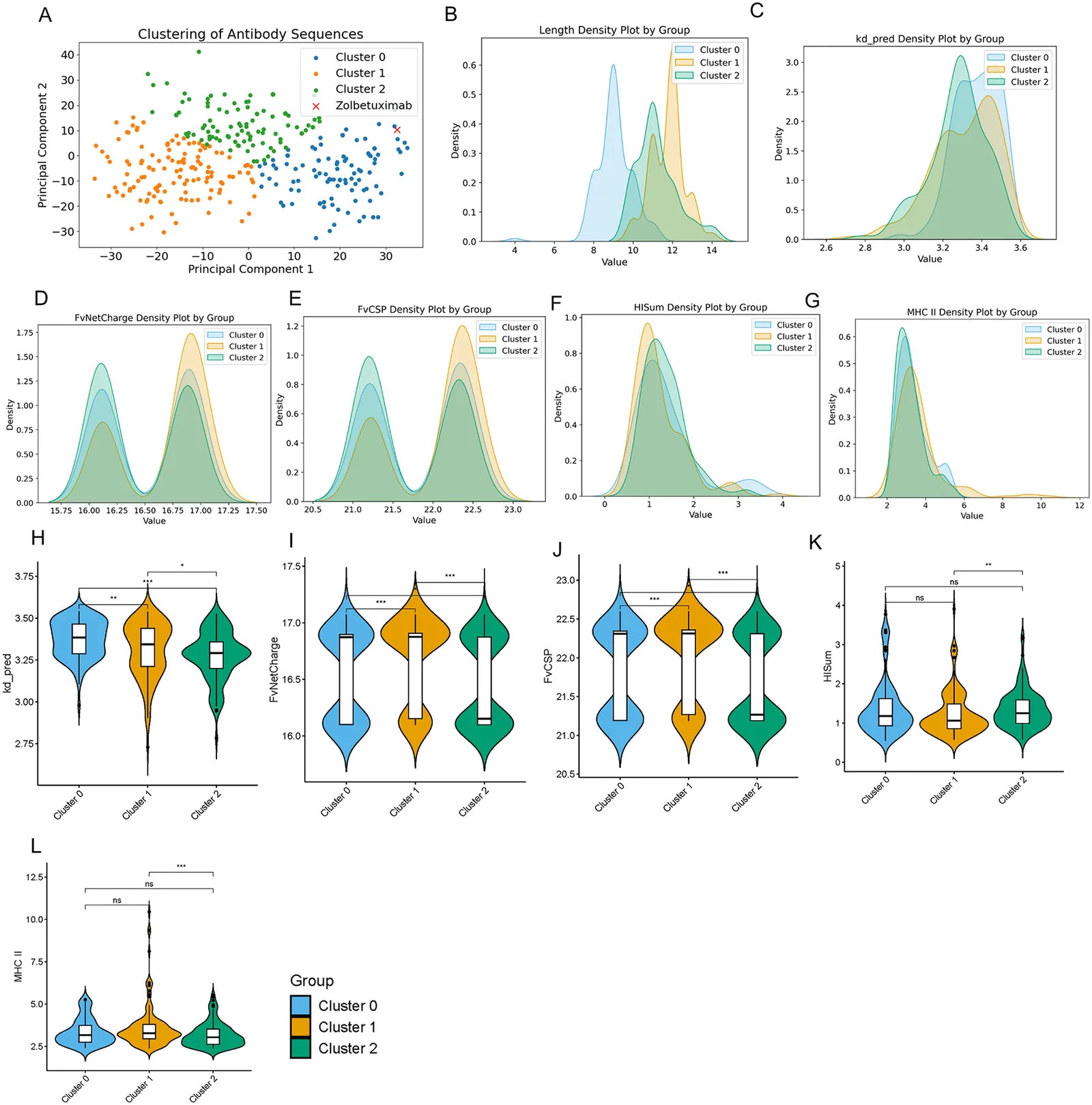
Clustering of ESM2-derived sequence features and cluster-specific property distributions. (A) Two-dimensional PCA visualization of antibody sequence embeddings generated by ESM2. (B–F) Density distributions of Kd_pred, FvNetCharge, FvCSP, HISum, and MHC II across Cluster0, Cluster1, and Cluster2. (G–K) Violin plots showing differences in these variables among clusters. Kd_pred, predicted dissociation constant; FvNetCharge, variable fragment net charge; FvCSP, variable fragment charge symmetry parameter; HISum, hydrophobicity index sum; MHC II, major histocompatibility complex class II score. *P < 0.05; **P < 0.01; ***P < 0.001; ns, not significant.

As shown in Fig 9A, the sequences formed three distinct clusters (Clusters 0, 1, and 2), represented by blue (n = 95), orange (n = 126), and green (n = 92) density points, respectively. The tight spatial distribution within each cluster reflects high feature similarity among its members, suggesting common antigen-binding profiles, structural architectures, or evolutionary origins. Notably, the CLDN18.2-targeting antibody zolbetuximab (red “×”) co-localized in Cluster 0, indicating strong sequence homology and functional relatedness with other cluster members.

Analysis of Cluster 0 revealed consistent trends across key sequence features (Fig 9B–9G): CDRH3 lengths concentrated at 10–12 residues; kd_Pred values clustered between 3.0-3.2, indicating similar solubility; FvNetCharge centered around 16.5-17.0; FvCSP peaked at 21.5-22.0; HISum values ranged from 1.0-1.5; and MHC II values distributed between 2.0-4.0.

To further validate the clustering, we compared key developability metrics across all clusters (Fig 9H–9L). Violin plots showed distinct distributions, with significant inter-cluster differences (P < 0.05) in kd_Pred, FvNetCharge, and FvCSP.

The high feature consistency within Cluster 0 provides mechanistic insight into zolbetuximab’s properties and offers a rational framework for designing and optimizing other CLDN18.2-targeting antibodies, highlighting the biological relevance and translational potential of this cluster.

### The LASSO model was used to quantify the contribution of different features and samples to the model

**Fig 10:**
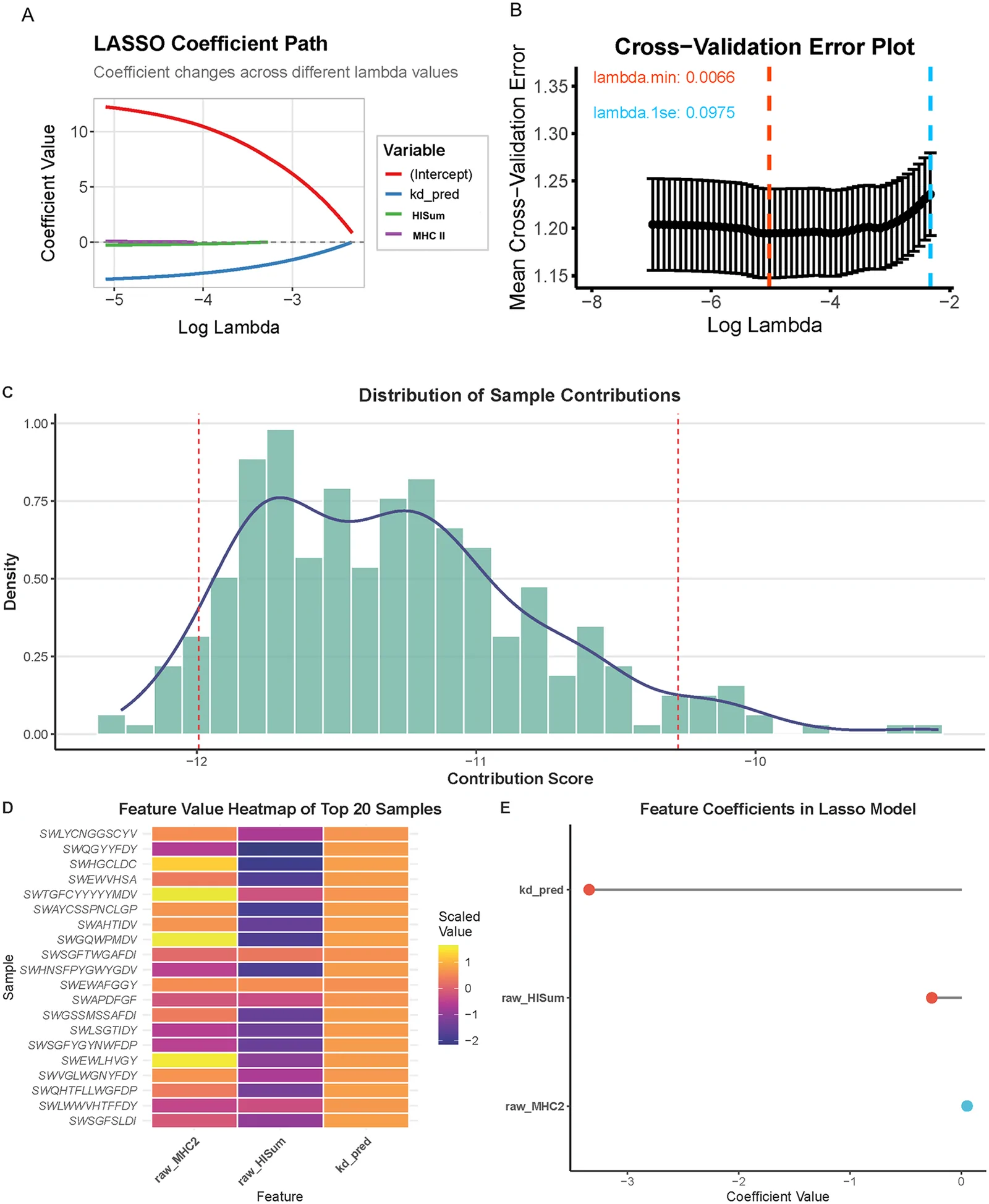
The LASSO model was used to quantify the contribution of different features and samples to the model. (A) The change paths of variable coefficients in the LASSO model under different lambda values. (B) Cross-validation errors of the LASSO model at different lambda values. (C) Distribution of sample contribution scores. (D) Heatmap of feature values in the top 20 samples. (E) Coefficients of individual features in the LASSO model, points of different colors represent various features, including kd_pred (red), HiSum (green), and MHC II (blue). kd_pred, predicted dissociation constant; HISum, hydrophobicity index sum; MHC, major histocompatibility complex II score.

To identify the most predictive features for the target variable, we applied the LASSO regression algorithm. As shown in Fig 10A and 10B, LASSO modulates the regularization parameter λ to control model complexity, selecting an optimal subset of features. Fig 10C presents the distribution of sample contribution values, which follows a left-skewed pattern ranging from approximately -12 to -10. This reflects the variation in individual sample contributions to the model’s prediction error, with the majority of samples clustering near -12 and a smaller subset exhibiting higher values. These insights help interpret sample roles within the model and highlight candidates warranting further study.

In Fig 10D, features are represented on a color scale from purple (low) to yellow (high), illustrating how LASSO achieves a balance between sparsity and predictive accuracy during model construction. The coefficient path in Fig 10E further visualizes the feature selection process. Together, these analyses clarify the primary factors driving model predictions.

Based on the LASSO results, we selected the top 20 sequences ranked by their regression coefficients. From these, seven sequences belonging to Cluster 0 (S4 Table) were chosen for subsequent validation and functional analysis.

### The potential structure of the antibody sequence and its complex structure with the antigen predicted by AF3

**Fig 11:**
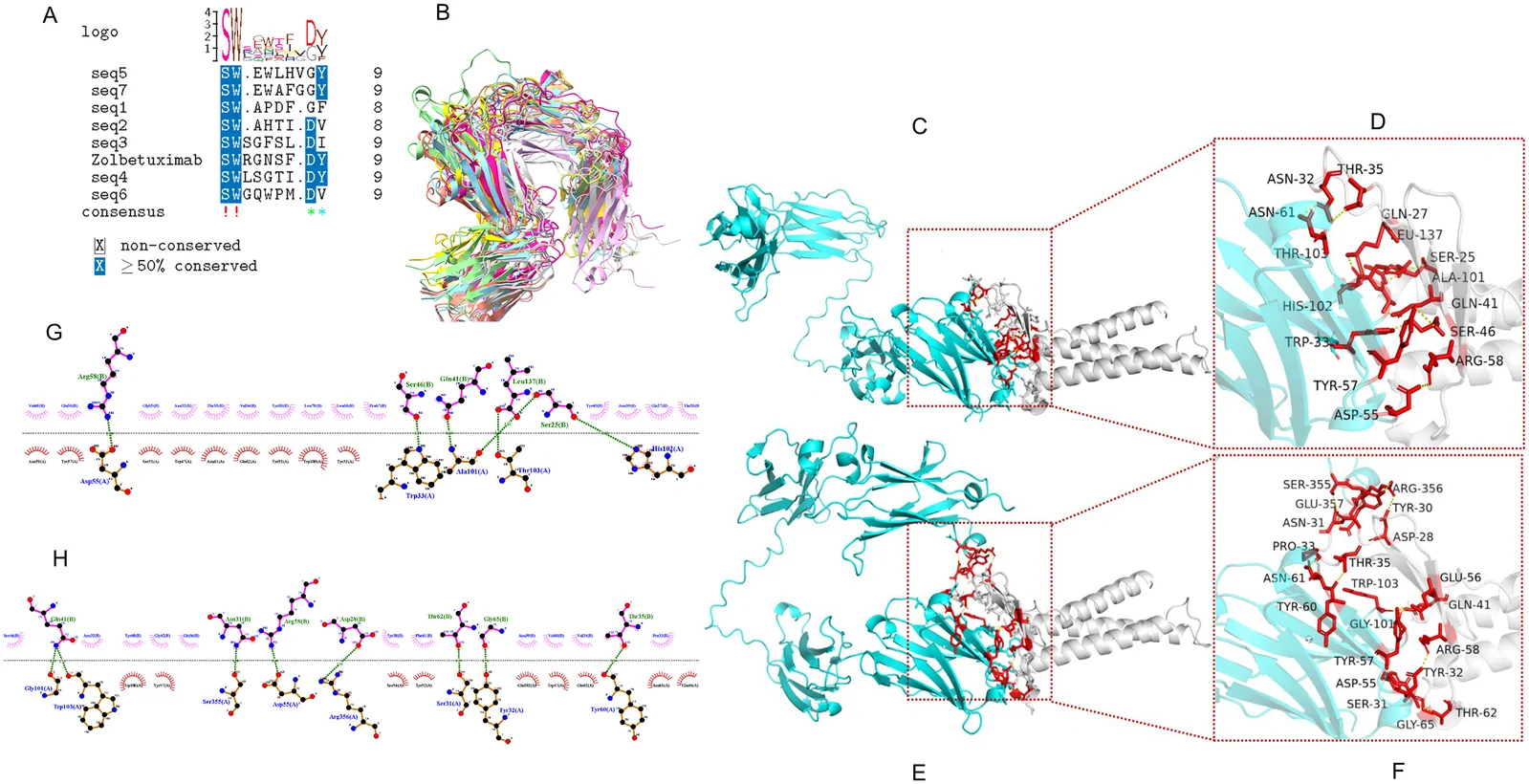
Structural assessment of representative designed CDRH3 candidates and their predicted complexes with CLDN18.2. (A) Multiple sequence alignment of representative designed CDRH3 sequences and the parental zolbetuximab CDRH3. Conserved residues are highlighted. (B) Structural superposition of representative designed antibody variable domains with the zolbetuximab-derived template. (C, E) AF3-predicted complex models of representative designed antibodies bound to the CLDN18.2 extracellular region. The generated CDRH3 sequences of seq2 and seq6 were grafted onto the zolbetuximab heavy-chain framework before complex modeling. (D, F) Zoomed-in views of the predicted paratope–epitope interfaces generated in PyMOL. The antibody is shown in cyan, the CLDN18.2 extracellular region is shown in gray, and residues located within 5 Å of the predicted interface are highlighted in red. These highlighted residues indicate spatially proximal interface residues in the AF3-predicted models, rather than experimentally validated binding determinants. (G, H) LigPlot-generated two-dimensional schematics summarizing potential hydrogen-bonding and hydrophobic contact patterns at the predicted interfaces.

To characterize shared features among the candidate antibodies, we first performed multiple sequence alignment of the seven selected CDRH3 sequences and generated sequence logos using the “msa” R package [29]. The generated CDRH3 sequences retained several residues commonly associated with antibody binding interfaces, including Trp, Phe, Tyr, Gly, Pro, and Asp, within central or terminal regions of the loop. These residues may contribute to local conformational variability, hydrophobic packing, or interface formation, although their functional roles require experimental validation. We subsequently predicted the three-dimensional structures of zolbetuximab and the seven candidate antibodies using AF3. The candidate models were superimposed onto the zolbetuximab reference structure based on Cα atoms using the MatchMaker module in UCSF Chimera [30]. The resulting RMSD values were within the predefined threshold of ≤ 2.0 Å (S5 Table), indicating that the designed candidates largely preserved the overall variable-domain architecture of the parental scaffold. Sequence and structural comparison further suggested high conformational similarity, particularly in the secondary-structure elements of the heavy chain (Fig 11A and 11B).

To further examine the predicted CLDN18.2-binding mode, we selected two representative candidates, seq2 and seq6, based on AF3 prediction confidence and structural consistency (S6A Fig and S6 and S7 Tables). The generated CDRH3 sequences were grafted onto the heavy-chain framework of zolbetuximab, and the resulting antibody models were used for AF3-based complex prediction with the extracellular region of CLDN18.2. In the predicted complex models, the designed CDRH3 loops were positioned near the CLDN18.2 extracellular region and were predicted to form local contacts with the antigen surface (Fig 11C–11F). To clarify the main paratope–epitope interface, zoomed-in views were generated in PyMOL by displaying antibody and antigen residues located within 5 Å of the predicted interface; these spatially proximal interface residues are highlighted in red in Fig 11D and 11F. For seq2, the predicted interface included residues such as ALA101, HIS102, and THR103, together with neighboring residues in the CLDN18.2 extracellular region, including GLN41, SER25, SER46, ARG58, and LEU137 (Fig 11C and 11D). For seq6, the predicted interface involved multiple spatially proximal residues, including GLY101, TRP103, GLN41, GLU56, and ARG58 (Fig 11E and 11F). These predicted contacts may contribute to the modeled antibody-antigen interface, but they should be interpreted as computational structural hypotheses rather than experimentally confirmed binding determinants.

To assess potential isoform selectivity, we further performed AF3-based comparative modeling of representative candidates against both CLDN18.1 and CLDN18.2 using multiple random seeds. This design allowed us to evaluate the robustness of AF3-predicted scores across independent modeling runs. The predicted metrics did not provide strong evidence for consistent CLDN18.2-over-CLDN18.1 discrimination (S6 Fig). These results suggest that isoform specificity remains unresolved and will require direct experimental cross-reactivity testing in future work.

To further assess the dynamic stability of the representative designs, 100 ns MD simulations were performed for Seq2 and Seq6 in complex with CLDN18.2. Both complexes remained dynamically stable without sustained dissociation or progressive unfolding. Seq2 maintained an average chain A-chain B interface distance of approximately 6.56 nm, whereas Seq6 showed a more compact interface with an average distance of approximately 5.47 nm. Stable temperature, pressure, and potential energy profiles further supported proper equilibration of the simulations. These results suggest that the selected AI-generated CDRH3 loops are compatible with the parental antibody framework within the simulated time scale. The full MD simulation profiles, including RMSD, RMSF, radius of gyration, temperature, pressure, potential energy, and interface distance, are provided in S7 and S8 Figs.

## 4. Discussion

This study presents cdrGPT, a GPT-2-based framework for scaffold-constrained CDRH3 sequence generation and prioritization in anti-CLDN18.2 antibodies. The framework integrates autoregressive sequence generation, multi-parameter in silico screening, and developability-aware candidate selection. By focusing on CDRH3 optimization within the zolbetuximab-derived anti-CLDN18.2 antibody scaffold, cdrGPT restricts the design space to a therapeutically relevant hypervariable region while preserving the broader structural context of the parental antibody [31].

The choice of a GPT-2-style autoregressive decoder was motivated by its suitability for modeling conditional sequence probabilities and generating variable-length sequences. This property is useful for CDRH3 design, where both residue composition and loop length contribute to antibody diversity. Pretraining on the Observed Antibody Space database enabled cdrGPT to learn statistical patterns of natural antibody repertoires, including residue preferences and CDRH3 length distributions. After two rounds of fine-tuning, the generated candidates showed improved convergence toward the zolbetuximab reference profile across several developability-related metrics.

A key feature of the framework is its integration of generation with multi-criteria candidate prioritization. Accordingly, the present evaluation should be interpreted as a downstream candidate-prioritization workflow rather than as a conventional supervised train/validation/test benchmark. Generated sequences were evaluated using FvNetCharge, FvCSP, HISum, MHC II minPR, AlphaBind-derived predicted binding ranking, and CDRH3 length, following the developability and immunogenicity-related criteria described in the Methods. These metrics were selected to assess complementary properties, including biophysical developability, immunogenicity-related risk, predicted binding potential, and sequence diversity. This evaluation strategy is not intended to replace experimental testing, but to reduce the candidate pool to a compact set of sequences with more favorable computational profiles for downstream validation.

To place cdrGPT in context, we compared it with AbLang and AntiBERTy under the same zolbetuximab-based CDRH3 grafting and property-assessment workflow (Fig 7). These models were selected as representative sequence-based antibody language-model baselines because they can be evaluated within the same sequence-level design and filtering framework [32,33]. In this controlled benchmark, cdrGPT showed a metric-dependent performance profile, with favorable HISum and FvNetCharge performance and the ability to generate variable-length CDRH3 sequences, but weaker MHC II minPR performance. These results suggest that cdrGPT should be interpreted as a scaffold-constrained, CDRH3-focused generation and prioritization framework rather than as a universally superior antibody design model.

The computational efficiency of cdrGPT should also be interpreted in this task-specific context. The model was trained using four NVIDIA A100 GPUs, and inference was performed on one NVIDIA A100 GPU. Under this setting, the generation and computational evaluation of 20,000 candidate CDRH3 sequences required approximately 4 h. This tractability reflects the restricted CDRH3 design space and retention of the parental antibody scaffold. However, because direct runtime comparisons with full-variable-region generative models, diffusion-based methods, or structure-based design pipelines were not performed, the reported efficiency should not be interpreted as general computational superiority.

Several limitations should be acknowledged. First, the model is shaped by its training data. Because the current training set was derived from OAS sequences selected for human or human-like antibody design, cdrGPT is most suitable for human or humanized IgG-class antibody design and may be less effective for distant non-human sequence spaces. Second, the immunogenicity assessment remains incomplete. MHC class II binding prediction provides a practical proxy for T-cell epitope-related risk, but it does not fully capture therapeutic antibody humanness, conformational epitopes, or other immune mechanisms. Future work should incorporate dedicated humanness assessment tools, such as BioPhi or OASis, together with broader immunogenicity evaluation strategies [34].

Third, the current binding and specificity assessments require cautious interpretation. AlphaBind, a recently reported domain-specific model for antibody–antigen binding affinity prediction and optimization, was used here only as a relative ranking tool anchored to the zolbetuximab reference [24]. Because its performance has not been specifically established for CLDN18.2 in this study, AlphaBind-derived scores should not be interpreted as calibrated affinity values or direct proof of binding accuracy. In addition, CLDN18.1 and CLDN18.2 differ in their first extracellular region encoded by alternative first exons, and isoform-specific recognition depends on this region [35,36]. Consistent with the AF3-based counter-screening of Seq2 and Seq6 against both isoforms, the current computational results did not establish robust CLDN18.2-over-CLDN18.1 discrimination. Therefore, computational prioritization alone cannot exclude potential CLDN18.1 cross-reactivity. Future studies should explicitly evaluate CLDN18.2 selectivity over CLDN18.1 using isoform-specific binding assays and, where possible, orthogonal computational modeling of both targets.

Fourth, CDRH3 grafting may affect the local paratope architecture, VH/VL orientation, and loop-scaffold interface. Although AlphaFold3-based structural modeling provides useful structural hypotheses, antibody–antigen complex prediction can be sensitive to sampling; therefore, representative candidates were assessed across multiple AF3 random seeds in the present study. Static structural predictions nevertheless do not replace dynamic stability assessment. The 100 ns molecular dynamics simulations performed in this study provided an initial assessment of scaffold compatibility and short-term complex stability, but additional structure-aware analyses and longer-timescale simulations would further help evaluate whether the designed CDRH3 loops preserve a stable binding-competent conformation.

Finally, the present findings remain computational and require experimental validation. The prioritized candidates should be tested for binding affinity, CLDN18.2/CLDN18.1 specificity, expression, stability, and functional activity using SPR or BLI, ELISA, cell-based binding assays, and downstream functional assays. Broader benchmarking against structure-aware or inverse-folding methods, such as AntiFold, diffusion-based and structure-aware generative approaches, such as RFdiffusion-style antibody design and CHAI-2-like methods, as well as Rosetta-style grafting methods, will also be important for defining the relative strengths and limitations of cdrGPT [37–41].

In summary, cdrGPT provides a computationally tractable framework for CDRH3-focused sequence generation and developability-aware prioritization within a fixed therapeutic antibody scaffold. The results support its feasibility as an in silico candidate-prioritization tool, while also highlighting the need for experimental validation, isoform-specific selectivity assessment, structural stability analysis, and broader benchmarking before its therapeutic utility can be fully established.

## Supporting information

S1 Fig Statistical data of Complementarity Determining Region Heavy Chain 3 (CDRH3) sequences collected from the Open Antibody Space (OAS) database. A. Density curve plot of the lengths of CDRH3 sequences. B. Violin plot box of the lengths of CDRH3 sequences. The observed median length is 13.
(DOCX)

S2 Fig Training Loss Analysis Diagram in Pre_CLDN18.2. (DOCX)

S3 Fig Training Loss Analysis Diagram in Pre_HER2. (DOCX)

S4 Fig Property distributions of generated CDRH3 sequences across three therapeutic antibody benchmark systems. (DOCX)

S5 Fig Success rates of developability-related parameters across three therapeutic antibody benchmark systems. (DOCX)

S6 Fig Score-organized AlphaFold 3 (AF3) random-seed profile plots for Seq2 and Seq6 modeled with CLDN18.1 and CLDN18.2. (DOCX)

S7 Fig Seq2 MD simulation profile. Seq2 underwent an initial structural relaxation but remained dynamically stable without sustained dissociation or progressive unfolding. The chain A-chain B interface distance fluctuated around approximately 6.56 nm, indicating maintenance of the antibody-antigen interface.
(DOCX)

S8 Fig Seq6 MD simulation profile. Seq6 showed a more compact and stable interaction pattern. The chain A-chain B interface distance was centered around approximately 5.47 nm, with only brief transient deviations and no sustained separation of the complex.
(DOCX)

S1 Table Evaluation of the models on predefined metrics. (DOCX)

S2 Table Hyperparameter configuration for pre-training and fine-tuning of cdrGPT. (DOCX)

S3 Table The evaluation of the models on predefined metrics. (DOCX)

S4 Table Multi-parameter evaluation of selected high-contribution CDRH3 candidate sequences. (CSV)

S5 Table Structural comparison of Zolbetuximab CLDN18.2 models. (DOCX)

S6 Table Prediction results of the AF3 complex structure. (DOCX)

S7 Table Prediction results of the AF3 monomer structure. (DOCX)
