# Supplementary material for: CLDN18.2 antibody design with protein language models: A deep learning optimization framework
Source: PLoS Comput Biol. 2026 Aug 20;22(8):e1014499. doi: 10.1371/journal.pcbi.1014499 (PMC13515015; doi:10.1371/journal.pcbi.1014499)
Supplement: Supplementary file 1 [file pcbi.1014499.s001.docx]

**S1 Fig.**

By training the prior model with CDRH3 sequences within this length interval, the model was enabled to effectively learn the characteristics of the CDRH3 sequence space within a similar length range, thereby enhancing the model's prediction and generalization abilities in this specific sequence space. A total of 637,887 non-repetitive CDRH3 sequences were finally obtained. S1_Fig. A shows the density distribution curve and violin plot of the lengths of these sequences. The analysis reveals that the median length is 13 amino acid residues.

S1 Fig. Statistical data of Complementarity Determining Region Heavy Chain 3 (CDRH3) sequences collected from the Open Antibody Space (OAS) database

A. Density curve plot of the lengths of CDRH3 sequences.

B. Violin plot box of the lengths of CDRH3 sequences. The observed median length is 13.
