# Supplementary material for: CLDN18.2 antibody design with protein language models: A deep learning optimization framework
Source: PLoS Comput Biol. 2026 Aug 20;22(8):e1014499. doi: 10.1371/journal.pcbi.1014499 (PMC13515015; doi:10.1371/journal.pcbi.1014499)
Supplement: Supplementary file 2 [file pcbi.1014499.s002.docx]

**S2 Fig.**

S2 Fig shows the loss function curve in the figure clearly delineates three critical phases of the model training process: In epoch 2, the loss value rapidly decreases, indicating the model's initial adaptation and quick learning of the training data; upon entering epoch 4, the rate of loss decline slows down as the model begins a more refined fitting and parameter optimization of the data; epoch 6, situated between epoch 4 and 8, marks the critical transition point where the model moves from stable learning to the potential onset of overfitting. During this phase, the model's fit to the training data is further enhanced, yet it also approaches the limits of its generalization capability. However, in epoch 8, there is a noticeable increase in the loss value, which may signal the beginning of overfitting to the training data. By incorporating the description of epoch 10 into the training loss analysis, we can gain a more comprehensive understanding of the model's behavior in the later stages of training, which is crucial for ensuring the model's stability and generalization ability. Through the analysis of these phases, we can more accurately grasp the model's learning progress and adjust training strategies in a timely manner to optimize the model's generalization capability.

S2 Fig. Training Loss Analysis Diagram in Pre_CLDN18.2.
