# Supplementary material for: CLDN18.2 antibody design with protein language models: A deep learning optimization framework
Source: PLoS Comput Biol. 2026 Aug 20;22(8):e1014499. doi: 10.1371/journal.pcbi.1014499 (PMC13515015; doi:10.1371/journal.pcbi.1014499)
Supplement: Supplementary file 4 [file pcbi.1014499.s004.docx]

**S4 Fig.**

**Cross-scenario generalization and comparative performance of CLDN18.2-specific antibody design models**

Cross-scenario validation of the modeling pipeline

To evaluate the cross-scenario applicability of the modeling pipeline, we selected three structurally resolved therapeutic antibody–antigen complexes from the Protein Data Bank: adalimumab, an anti-TNF-α antibody (PDB: 2AZ5); bevacizumab, an anti-VEGFA antibody (PDB: 5DN2); and panitumumab, an anti-EGFR antibody (PDB: 1M17). These systems were chosen to represent different antigen classes, including cytokines, growth factors, and cell-surface receptors. Using the same pipeline applied in the CLDN18.2-focused study, CDRH3 sequences were generated for each antibody, grafted onto the corresponding antibody framework, and evaluated using multiple biophysical and structural criteria.

The distributional characteristics of the evaluated properties are shown in S4 Fig. Across the three antibody systems, the generated sequences showed antibody-dependent distribution patterns for FvCSP, FvNetCharge, HISum, minPR, sequence length, and kd_pred. These results indicate that the pipeline remained sensitive to the sequence and structural context of each antibody framework rather than producing a uniform profile across all systems. This antibody-dependent variability is expected, given that CDRH3 replacement can have different effects depending on the scaffold and antigen context.

S4 Fig. Property distributions of generated CDRH3 sequences across three therapeutic antibody benchmark systems.

Density distributions of FvCSP, FvNetCharge, HISum, sequence length, MHC II minPR, and kd_pred are shown for CDRH3 sequences generated and evaluated under the adalimumab, bevacizumab, and panitumumab benchmark settings. These antibody systems represent distinct scaffold and antigen contexts, including anti-TNF-α, anti-VEGFA, and anti-EGFR antibodies. The distributions show antibody-dependent variation in sequence- and developability-related properties, indicating that the pipeline remains sensitive to the scaffold and antigen context rather than producing a uniform property profile across different antibody systems.
