# Supplementary material for: CLDN18.2 antibody design with protein language models: A deep learning optimization framework
Source: PLoS Comput Biol. 2026 Aug 20;22(8):e1014499. doi: 10.1371/journal.pcbi.1014499 (PMC13515015; doi:10.1371/journal.pcbi.1014499)
Supplement: Supplementary file 5 [file pcbi.1014499.s005.docx]

**S5 Fig.**

**Cross-scenario generalization and comparative performance of CLDN18.2-specific antibody design models**

To further summarize the benchmark results, we calculated success rates for key parameters, including FvNetCharge, FvCSP, HISum, minPR, and kd_pred (S5_Fig.). The success-rate profiles varied across antibody systems. Adalimumab showed relatively high success rates for FvCSP and minPR, bevacizumab showed the highest success rate for FvNetCharge, and panitumumab showed the highest success rate for kd_pred. In contrast, HISum success rates were consistently high across all three antibodies. These findings suggest that the pipeline can identify favorable CDRH3 candidates under multiple antibody settings, but the performance is strongly metric- and scaffold-dependent.

Taken together, the analyses in S4_Fig. and S5_Fig support the cross-scenario applicability of the modeling pipeline at the level of computational candidate assessment. At the same time, the observed variability across antibodies highlights the technical challenge of generalizing CDRH3 grafting and prioritization across structurally and functionally distinct antibody-antigen systems.

S5 Fig. Success rates of developability-related parameters across three therapeutic antibody benchmark systems.

Success rates for FvNetCharge, FvCSP, HISum, MHC II minPR, and kd_pred are shown for the adalimumab, bevacizumab, and panitumumab benchmark systems. The three antibodies displayed distinct metric-specific success-rate profiles: adalimumab showed relatively favorable performance for FvCSP and MHC II minPR, bevacizumab showed the highest success rate for FvNetCharge, and panitumumab showed the highest success rate for kd_pred. HISum success rates were consistently high across all three systems. These results suggest that the pipeline can identify computationally favorable CDRH3 candidates across different antibody settings, while its performance remains metric- and scaffold-dependent.
