# Supplementary material for: CLDN18.2 antibody design with protein language models: A deep learning optimization framework
Source: PLoS Comput Biol. 2026 Aug 20;22(8):e1014499. doi: 10.1371/journal.pcbi.1014499 (PMC13515015; doi:10.1371/journal.pcbi.1014499)
Supplement: Supplementary file 6 [file pcbi.1014499.s006.docx]

**S6 Fig.**

**AlphaFold 3-based comparative modeling of Seq2 and Seq6 with CLDN18.1 and CLDN18.2 across random seeds**

To evaluate the isoform selectivity of the CLDN18.2-directed antibody candidates and the stability of AlphaFold 3 (AF3) predictions across random seeds, we performed comparative modeling using Seq2 and Seq6. Each candidate was modeled in complex with the extracellular region of CLDN18.2 or the closely related isoform CLDN18.1. Independent predictions were generated using three pre-defined random integer seeds: 396, 479, and 875.

The results are presented according to six AF3-derived metrics: ipTM, pTM, ranking score, Fraction Disordered, mean chain ipTM, and mean chain pTM. Each panel shows the corresponding metric across random seeds and compares the predicted complexes formed by Seq2 or Seq6 with either CLDN18.1 or CLDN18.2.

The AF3 scores for Seq2 and Seq6 showed some seed-dependent variation, indicating that individual predictions can be affected by the random seed. However, these variations did not alter the overall interpretation. Although CLDN18.2-associated predictions were slightly higher in some metrics or seed runs, this pattern was not consistently observed across all scores and seeds. Overall, the model-quality and chain-level confidence scores for Seq2 and Seq6 were comparable between CLDN18.1 and CLDN18.2. These results indicate that the current AF3 analysis alone is insufficient to establish clear CLDN18.2 isoform specificity. Thus, these sequences should be considered prioritized CLDN18.2-directed antibody candidates, while their true CLDN18.2 selectivity and potential cross-reactivity with CLDN18.1 require further experimental validation.

S6 Fig. Score-organized AlphaFold 3 (AF3) random-seed profile plots for Seq2 and Seq6 modeled with CLDN18.1 and CLDN18.2.

Seq2 and Seq6 were modeled in complex with the extracellular region of either CLDN18.1 or CLDN18.2 using AF3 with three random seeds: 396, 479, and 875. The six panels are organized by AF3-derived metric: ipTM, pTM, ranking score, Fraction Disordered, mean chain ipTM, and mean chain pTM. The x axis denotes the random seed. Solid and dashed lines indicate Seq2 and Seq6, respectively; blue circles and orange squares indicate CLDN18.1 and CLDN18.2, respectively. Points represent individual AF3 predictions; connecting lines are used only to guide the eye and do not indicate continuous trends.
