# Supplementary material for: CLDN18.2 antibody design with protein language models: A deep learning optimization framework
Source: PLoS Comput Biol. 2026 Aug 20;22(8):e1014499. doi: 10.1371/journal.pcbi.1014499 (PMC13515015; doi:10.1371/journal.pcbi.1014499)
Supplement: Supplementary file 7 [file pcbi.1014499.s007.docx]

**S7 Fig.**

**Molecular dynamics simulation analysis of Seq2 and Seq6 antibody-antigen complexes**

Molecular dynamics (MD) simulation profiles of two representative designed antibody-CLDN18.2 antigen complexes, Seq2 and Seq6, over 100 ns simulations. The analyses include RMSD, RMSF, radius of gyration (Rg), temperature, pressure, potential energy, and chain A–chain B interface distance. Seq2 maintained the antibody–antigen interface with an average interface distance of approximately 6.56 nm, whereas Seq6 showed a more compact interface with an average distance of approximately 5.47 nm. Both systems showed stable temperature, pressure, and potential energy profiles, supporting proper equilibration and overall structural stability of the simulated complexes.

S7 Fig. Seq2 MD simulation profile. Seq2 underwent an initial structural relaxation but remained dynamically stable without sustained dissociation or progressive unfolding. The chain A-chain B interface distance fluctuated around approximately 6.56 nm, indicating maintenance of the antibody-antigen interface.
