# Supplementary material for: CLDN18.2 antibody design with protein language models: A deep learning optimization framework
Source: PLoS Comput Biol. 2026 Aug 20;22(8):e1014499. doi: 10.1371/journal.pcbi.1014499 (PMC13515015; doi:10.1371/journal.pcbi.1014499)
Supplement: Supplementary file 8 [file pcbi.1014499.s008.docx]

**S8 Fig.**

**Molecular dynamics simulation analysis of Seq2 and Seq6 antibody-antigen complexes**

S8 Fig. Seq6 MD simulation profile. Seq6 showed a more compact and stable interaction pattern. The chain A-chain B interface distance was centered around approximately 5.47 nm, with only brief transient deviations and no sustained separation of the complex.
