# Supplementary material for: CLDN18.2 antibody design with protein language models: A deep learning optimization framework
Source: PLoS Comput Biol. 2026 Aug 20;22(8):e1014499. doi: 10.1371/journal.pcbi.1014499 (PMC13515015; doi:10.1371/journal.pcbi.1014499)
Supplement: Supplementary file 9 [file pcbi.1014499.s009.docx]

**S1 Table.**

To ascertain the optimal hyperparameters of the model, we conducted a systematic exploration of the parameters across various model configurations. S1_Table presents the comprehensive list of hyperparameters examined, with each parameter's impact rigorously evaluated.In the pre-trained models, we focused on assessing six key hyperparameters: the number of layers (Num layers), the embedding dimension (N_embedding), the layer size (Layer size), the number of attention heads (N_head), the number of parameters (N_params), and the number of training epochs (Epoch). All models underwent training for 10 epochs.Number of Layers: Models with a greater number of layers demonstrated superior loss convergence in each epoch. However, training time and memory requirements increased proportionally with the number of layers. We selected configurations with 8 and 3 layers to achieve the best trade-off between learning capability and resource utilization. Embedding Dimension: Increasing the embedding dimension enhances the model's representational capacity but also enlarges the model size. We opted for an embedding dimension of 256 to balance representational ability with computational feasibility. Layer Size: The layer size dictates the number of neurons in each layer, influencing the model's learning capacity and complexity. We chose a layer size of 512 to ensure the model possesses sufficient capacity to learn complex features. Number of Attention Heads: For the GPT model, increasing the number of attention heads can reduce loss to a certain extent, but this benefit may be offset by a significant increase in training time. Consequently, we adopted a configuration with 8 attention heads. Number of Parameters: The number of parameters serves as a critical indicator of the model's complexity. Generally, a larger number of parameters implies greater expressive power but may also lead to overfitting. We selected configurations with varying numbers of parameters to ensure the model strikes a balance between performance and generalization capability. The selection of these hyperparameters reflects a deliberate trade-off between model performance, training efficiency, and hardware constraints, ensuring optimal scalability and reproducibility in downstream applications. Through the systematic exploration and evaluation of these hyperparameters, we were able to identify the model configuration that best meets the requirements of our task.

**S1 Table. Evaluation of the models on predefined metrics**

| **Model** | **Num layers** | **N_embedding** | **Layer size** | **N_head** | **N_params** | **Epoch** |
| --- | --- | --- | --- | --- | --- | --- |
| GPT | 8 | 256 | - | 8 | 6.38*10^6^ | 10 |
| GRU-L8 | 8 | 256 | 512 | - | 1.22*10^7^ | 10 |
| GRU-L3 | 3 | 256 | 512 | - | 4.35*10^6^ | 10 |

GRU-L3 :3-layer Gated Recurrent Unit; GRU-L8:8-layer Gated Recurrent Unit
