# Supplementary material for: CLDN18.2 antibody design with protein language models: A deep learning optimization framework
Source: PLoS Comput Biol. 2026 Aug 20;22(8):e1014499. doi: 10.1371/journal.pcbi.1014499 (PMC13515015; doi:10.1371/journal.pcbi.1014499)
Supplement: Supplementary file 10 [file pcbi.1014499.s010.docx]

**S2_Table.**

Batch Size and Learning Rate:

Pre-training: A per-GPU batch size of 64 was used, or a total batch size of 1024 in distributed training (with gradient accumulation over 4 steps). The learning rate was set to 1e-4, with a linear warm-up applied over the first 10% of training steps.

Fine-tuning: A per-GPU batch size of 32 was used, with a total distributed batch size of 256 (gradient accumulation over 2 steps). The initial learning rate was 5e-5, decayed gradually to 1e-6 using a cosine annealing schedule.

Optimizer and Weight Decay:

Both pre-training and fine-tuning used the AdamW optimizer with β₁=0.9, β₂=0.999, and ε=1e-8. A weight decay coefficient of 0.01 was applied throughout. During fine-tuning, weight decay was applied only to trainable parameters to avoid unnecessary regularization on frozen parameters.

Training Epochs and Early Stopping:

Pre-training was conducted for 100 epochs and fine-tuning for 10 epochs. A dynamic early stopping mechanism was adopted: training was halted if no significant reduction (defined as a relative improvement lower than 1e-4) in validation loss was observed over 5 consecutive epochs, and the best-performing checkpoint was restored.

**S2_Table. Hyperparameter configuration for pre-training and fine-tuning of cdrGPT.**

| **Parameter Category** | **Pretraining Phase** | **Fine-tuning Phase** |
| --- | --- | --- |
| **Computational Resource** | 4 × NVIDIA A100 40GB | 4 × NVIDIA A100 40GB |
| **Training Duration** | ~120 hours | ~15 hours |
| **Global Batch Size** | 1024 | 256 |
| **Per-GPU Batch Size** | 64 | 32 |
| **Gradient Accumulation Steps** | 4 | 2 |
| **Initial Learning Rate** | 1e-4 | 5e-5 |
| **Learning Rate Schedule** | Linear warmup (first 10% of steps) | Cosine annealing to 1e-6 |
| **Optimizer** | AdamW (β₁=0.9, β₂=0.999, ε=1e-8) | AdamW (β₁=0.9, β₂=0.999, ε=1e-8) |
| **Weight Decay** | 0.01 | 0.01 (applied only to trainable parameters) |
| **Total Epochs** | 100 | 10 |
| **Early Stopping Criteria** | Patience: 5 Epochs, Δmin: 1e-4 | Patience: 5 Epochs, Δmin: 1e-4 |
