# Supplementary material for: CLDN18.2 antibody design with protein language models: A deep learning optimization framework
Source: PLoS Comput Biol. 2026 Aug 20;22(8):e1014499. doi: 10.1371/journal.pcbi.1014499 (PMC13515015; doi:10.1371/journal.pcbi.1014499)
Supplement: Supplementary file 11 [file pcbi.1014499.s011.docx]

**S3 Table.**

To evaluate the generative capability of the models, this study selected three basic metrics, namely uniqueness, novelty, and diversity. The metric values of the pre-trained model (pre_model), baseline model (OAS_model), and fine-tuned CLDN18.2 (FT_CLDN18.2) and HER2 (FT_HER2) models are presented in S3_Table.

The sequences generated by the pre-trained model exhibit extremely high uniqueness (1.000) and novelty (0.999); while the sequences generated by the baseline model also show high uniqueness (1.000), its novelty is 0, this is because all sequences of the baseline model are sampled from the training set, resulting in no novelty. Both two fine-tuned models have relatively low diversity metrics, with values of 4.741 and 5.459 respectively. In conclusion, the results demonstrate that the pre-trained model can generate CDRH3 (complementarity-determining region 3) sequences with both high uniqueness and diversity, and is capable of producing novel sequences not present in the training set.

**S3 Table. The evaluation of the models on predefined metrics.**

| Model | Uniqueness | Novelty | Diversity |
| --- | --- | --- | --- |
| OAS_model | 1.000 | 0.000 | 9.975 |
| Pre_model | 1.000 | 0.999 | 9.984 |
| FT_ CLDN18.2 | 0.992 | 1.000 | 4.741 |
| FT_ HER2 | 1.000 | 1.000 | 5.459 |

Note: Both FT_CLDN18.2 and FT_HER2 are agent models trained for multiple desirable properties, which include the affinity for CLDN18.2 and HER2, respectively, as well as viscosity, clearance, and immunogenicity. Among them, CLDN18.2 refers to Claudin 18.2 (a tight junction protein), and HER2 stands for human epidermal growth factor receptor
