# Supplementary material for: CLDN18.2 antibody design with protein language models: A deep learning optimization framework
Source: PLoS Comput Biol. 2026 Aug 20;22(8):e1014499. doi: 10.1371/journal.pcbi.1014499 (PMC13515015; doi:10.1371/journal.pcbi.1014499)
Supplement: Supplementary file 12 [file pcbi.1014499.s013.docx]

**S5 Table.**

We conducted sequence alignment of the 7 filtered sequences with Zolbetuximab, and the results are as follows:

Sequence Alignment Score: The sequence alignment scores for all comparison objects are relatively high, ranging from 2296.6 to 2329.2, indicating a high degree of sequence similarity among these models.

RMSD of Pruned Atom Pairs: The RMSD values of the pruned atom pairs are relatively low, from 0.210 to 1.466 Å. This suggests that after pruning, these models exhibit a high degree of similarity in local structure. Notably, the seq2 and seq3 have particularly low RMSD values of 0.381 Å and 0.347 Å, respectively, indicating an extremely high degree of local structural similarity.

RMSD of All Atom Pairs: The RMSD values of all atom pairs are relatively high, ranging from 4.592 to 10.725 Å, indicating that there are certain differences in the overall structure among these models. The seq5 and seq6 have particularly high overall RMSD values of 10.725 Å and 10.144 Å, respectively, indicating significant differences in overall structure.

Notes: The notes in the table briefly describe the characteristics of each comparison result, aiding in a quick understanding of the similarities and differences between each model.

In conclusion, despite the high degree of sequence similarity among these models, there are certain differences in their overall structure. In particular, certain models, such as seq2 and seq3, exhibit a high degree of similarity in local structure, while other models show significant differences in overall structure.

**S5 Table. Structural Comparison of Zolbetuximab CLDN18.2 Models**

| Seq | Seq | | Sequence Alignment Score | Number of Pruned Atom Pairs | RMSD of Pruned Atom Pairs (Å) | RMSD of All Atom Pairs (Å) |
| --- | --- | --- | --- | --- | --- | --- |
| Zolbetuximab | seq1 | | 2315.7 | 71 | 1.331 | 5.872 |
| Zolbetuximab | Seq2 | | 2312.8 | 211 | 0.381 | 8.786 |
| Zolbetuximab | Seq3 | | 2329.2 | 208 | 0.347 | 9.122 |
| Zolbetuximab | Seq4 | | 2303.5 | 142 | 1.258 | 4.592 |
| Zolbetuximab | Seq5 | | 2320.8 | 146 | 0.844 | 10.725 |
| Zolbetuximab | | Seq6 | 2304.9 | 39 | 1.466 | 10.144 |
| Zolbetuximab | | Seq7 | 2296.6 | 213 | 0.210 | 8.669 |
