# Supplementary material for: CLDN18.2 antibody design with protein language models: A deep learning optimization framework
Source: PLoS Comput Biol. 2026 Aug 20;22(8):e1014499. doi: 10.1371/journal.pcbi.1014499 (PMC13515015; doi:10.1371/journal.pcbi.1014499)
Supplement: Supplementary file 13 [file pcbi.1014499.s014.docx]

**S6 Table. Prediction results of the AF3 complex structure.**

| **Com** | **chain_iptm** | **chain_pair_iptm.1** | **chain_pair_iptm.2** | **chain_pair_pae_min.1** | **chain_pair_pae_min.2** | **chain_ptm** | **fraction_disordered** | **iptm** | **num_recycles** | **ptm** | **ranking_score** | **pLDDT (chainA-B)** |
| --- | --- | --- | --- | --- | --- | --- | --- | --- | --- | --- | --- | --- |
| **1** | **0.36,0.36** | **0.44,0.36** | **0.36,0.63** | **0.76,8.43** | **8.87,0.76** | **0.44，0.63** | **0.04** | **0.36** | **10** | **0.39** | **0.39** | **75.9, 63.7** |
| **2** | **0.45,0.45** | **0.44,0.45** | **0.45,0.62** | **0.76,7.41** | **7.67,0.76** | **0.44,0.62** | **0.03** | **0.45** | **10** | **0.41** | **0.46** | **77.6, 65.4** |
| **3** | **0.34,0.34** | **0.43,0.34** | **0.43,0.61** | **0.76,9.81** | **10.57,0.76** | **0.43,0.61** | **0.03** | **0.34** | **10** | **0.39** | **0.36** | **76.6. 63.3** |
| **4** | **0.33,0.33** | **0.44,0.33** | **0.33,0.62** | **0.76,9.61** | **10.46,0.76** | **0.44,0.62** | **0.03** | **0.33** | **10** | **0.38** | **0.35** | **76.4, 63.4** |
| **5** | **0.3,0.3** | **0.44,0.3** | **0.3,0.63** | **0.76,10.2** | **10.13,0.76** | **0.44,0.63** | **0.03** | **0.3** | **10** | **0.38** | **0.33** | **77.0, 63.6** |
| **6** | **0.38,0.38** | **0.44,0.38** | **0.38,0.63** | **0.76,7.67** | **8.16,0.76** | **0.44,0.63** | **0.03** | **0.38** | **10** | **0.39** | **0.4** | **76.6, 64.5** |
| **7** | **0.35,0.35** | **0.43,0.35** | **0.35,0.64** | **0.76,9.19** | **9.22,0.76** | **0.43,0.64** | **0.03** | **0.35** | **10** | **0.39** | **0.38** | **76.2, 63.8** |
| **ZOL** | **0.36,0.36** | **0.44,0.36** | **0.36,0.63** | **0.76,8.43** | **8.87,0.76** | **0.43,0.63** | **0.04** | **0.36** | **10** | **0.39** | **0.39** | **77.9, 64.9** |

chain_iptm: Intra-chain iPTM (Interaction Score-corrected Prediction Template Model), used to evaluate the interaction score within a single chain.

chain_pair_iptm: Chain pair iPTM, used to evaluate the interaction score between different chains.

chain_pair_pae_min: Minimum PAE (Predicted Alignment Error) of the chain pair, used to evaluate the alignment error between different chains.

chain_ptm: Chain PTM (Prediction Template Model) score, used to evaluate the PTM score of a single chain.

fraction_disordered: Proportion of disordered regions, representing the proportion of disordered regions in the prediction.

has_clash: Existence of a clash, indicating whether there is an atomic clash in the prediction model.

iptm: Score of the prediction template model with interaction score correction.

num_recycles: Number of cycles, representing the number of iterative calculations of the model. In this study, the value is 10.0, indicating that 10 iterations were performed.

ptm: Prediction Template Model score, used to evaluate the PTM score of the entire model.

ranking_score: Ranking score, used to rank different prediction models.

pLDDT：Predicted Local Distance Difference Test of chain A and chain B
