# Supplementary material for: CLDN18.2 antibody design with protein language models: A deep learning optimization framework
Source: PLoS Comput Biol. 2026 Aug 20;22(8):e1014499. doi: 10.1371/journal.pcbi.1014499 (PMC13515015; doi:10.1371/journal.pcbi.1014499)
Supplement: Supplementary file 14 [file pcbi.1014499.s015.docx]

**S7 Table. Prediction results of the AF3 monomer structure.**

| **seq** | **chain_iptm** | **chain_pair_iptm.1** | **chain_pair_pae_min** | **chain_ptm** | **fraction_disordered** | **iptm** | **num_recycles** | **ptm** | **ranking_score** |
| --- | --- | --- | --- | --- | --- | --- | --- | --- | --- |
| **1** | **Null** | **0.48** | **0.76** | **0.48** | **0.05** | **Null** | **10** | **0.48** | **0.51** |
| **2** | **Null** | **0.48** | **0.76** | **0.48** | **0.05** | **Null** | **10** | **0.48** | **0.51** |
| **3** | **Null** | **0.48** | **0.76** | **0.48** | **0.05** | **Null** | **10** | **0.48** | **0.51** |
| **4** | **Null** | **0.49** | **0.76** | **0.49** | **0.05** | **Null** | **10** | **0.49** | **0.51** |
| **5** | **Null** | **0.48** | **0.76** | **0.48** | **0.05** | **Null** | **10** | **0.48** | **0.51** |
| **6** | **Null** | **0.48** | **0.76** | **0.48** | **0.05** | **Null** | **10** | **0.48** | **0.51** |
| **7** | **Null** | **0.48** | **0.76** | **0.48** | **0.05** | **Null** | **10** | **0.48** | **0.5** |
| Zoltuximab | **Null** | **0.49** | **0.76** | **0.49** | **0.04** | **Null** | **10** | **0.49** | **0.51** |

chain_iptm: Intra-chain iPTM (Interaction Score-corrected Prediction Template Model), used to evaluate the interaction score within a single chain.

chain_pair_iptm: Chain pair iPTM, used to evaluate the interaction score between different chains.

chain_pair_pae_min: Minimum PAE (Predicted Alignment Error) of the chain pair, used to evaluate the alignment error between different chains.

chain_ptm: Chain PTM (Prediction Template Model) score, used to evaluate the PTM score of a single chain.

fraction_disordered: Proportion of disordered regions, representing the proportion of disordered regions in the prediction.

has_clash: Existence of a clash, indicating whether there is an atomic clash in the prediction model.

iptm: Score of the prediction template model with interaction score correction.

num_recycles: Number of cycles, representing the number of iterative calculations of the model. In this study, the value is 10.0, indicating that 10 iterations were performed.

ptm: Prediction Template Model score, used to evaluate the PTM score of the entire model.

ranking_score: Ranking score, used to rank different prediction models.

pLDDT：Predicted Local Distance Difference Test of chain A and chain B
