# Supplementary material for: CLDN18.2 antibody design with protein language models: A deep learning optimization framework
Source: PLoS Comput Biol. 2026 Aug 20;22(8):e1014499. doi: 10.1371/journal.pcbi.1014499 (PMC13515015; doi:10.1371/journal.pcbi.1014499)
Supplement: Supplementary file 15 [file pcbi.1014499.s003.docx]

**S3 Fig.**

S3 Fig. Training Loss Analysis Diagram in Pre_HER2
